# Going Beyond the Carothers, Flory and Stockmayer Equation by Including Cyclization Reactions and Mobility Constraints

**DOI:** 10.3390/polym13152410

**Published:** 2021-07-22

**Authors:** Lies De Keer, Paul H. M. Van Steenberge, Marie-Françoise Reyniers, Dagmar R. D’hooge

**Affiliations:** 1 Laboratory for Chemical Technology (LCT), Department of Materials, Textiles and Chemical Engineering, Faculty of Engineering and Architecture, Ghent University, Technologiepark 125, 9052 Ghent, Belgium; lies.dekeer@ugent.be (L.D.K.); paul.vansteenberge@ugent.be (P.H.M.V.S.); mariefrancoise.reyniers@ugent.be (M.-F.R.); 2 Centre for Textiles Science and Engineering (CTSE), Department of Materials, Textiles and Chemical Engineering, Faculty of Engineering and Architecture, Ghent University, Technologiepark 70a, 9052 Ghent, Belgium

**Keywords:** polymer networks, kinetic Monte Carlo, step-growth polymerization, diffusional limitations, ring formation

## Abstract

A challenge in the field of polymer network synthesis by a step-growth mechanism is the quantification of the relative importance of inter- vs. intramolecular reactions. Here we use a matrix-based kinetic Monte Carlo (*k*MC) framework to demonstrate that the variation of the chain length distribution and its averages (e.g., number average chain length *x_n_*), are largely affected by intramolecular reactions, as mostly ignored in theoretical studies. We showcase that a conventional approach based on equations derived by Carothers, Flory and Stockmayer, assuming constant reactivities and ignoring intramolecular reactions, is very approximate, and the use of asymptotic limits is biased. Intramolecular reactions stretch the functional group (FG) conversion range and reduce the average chain lengths. In the likely case of restricted mobilities due to diffusional limitations because of a viscosity increase during polymerization, a complex *x_n_* profile with possible plateau formation may arise. The joint consideration of stoichiometric and non-stoichiometric conditions allows the validation of hypotheses for both the intrinsic and apparent reactivities of inter- and intramolecular reactions. The *k*MC framework is also utilized for reverse engineering purposes, aiming at the identification of advanced (pseudo-)analytical equations, dimensionless numbers and mechanistic insights. We highlight that assuming average molecules by equally distributing A and B FGs is unsuited, and the number of AB intramolecular combinations is affected by the number of monomer units in the molecules, specifically at high FG conversions. In the absence of mobility constraints, dimensionless numbers can be considered to map the time variation of the fraction of intramolecular reactions, but still, a complex solution results, making a *k*MC approach overall most elegant.

## 1. Introduction

**Figure 1:**
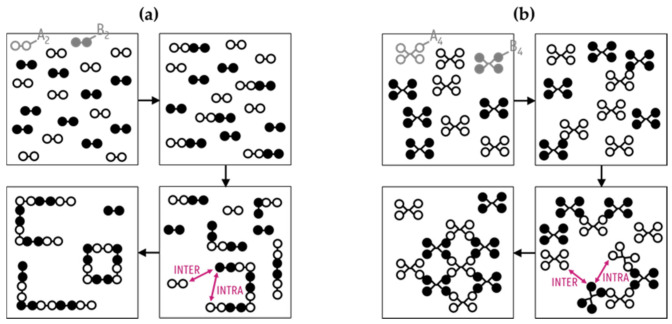
Principle of step-growth polymerization for (**a**) two bifunctional monomers (A_2_: white and B_2_: black) and (**b**) two multifunctional monomers (A_4_: white and B_4_: black); inter- and intramolecular bond formation are highlighted by purple arrows.

Step-growth polymerization involves gradual ligation reactions between either bifunctional or multifunctional components with the possible liberation of small molecules, such as water and alcohol [1]. In the former case of only bifunctional moieties, one refers to linear step-growth polymerization (e.g., monomeric reactants A_2_ and B_2_; Figure 1a), whereas in the latter case with multifunctional moieties, one considers the term network step-growth polymerization (e.g., monomeric reactants A_4_ and B_4_; Figure 1b). Each time one reacted functional group pair (“AB”) is formed, the remaining functionalities allow (in principle) for further growth so that in a theoretical limit, one large molecule results. In the presence of byproduct formation, one refers to polycondensation, with many examples both for natural and synthetic polymers [2,3,4,5]. The synthesis of the first synthetic step-growth polymeric material, i.e., bakelite, which was introduced in 1907 by Leo Baekeland, considered phenol and formaldehyde groups as the building blocks [6]. The largest-volume polymers made via step-growth polymerization are polyesters and polyamides, with main applications as the production of fibers, wire coatings and composites [1,7,8,9,10,11]. Well-known examples are polyester plastic bottles made from poly(ethylene terephthalate) and polyamide nylon 6,6. Furthermore, polycarbonates are used as digital-storage-media substrates and for electronic devices due to their transparency and high toughness [1,12]. Other commercial examples are polyurethanes and cured epoxy resins [11,13].

For the characterization of step-growth polymerization, the main focus is on the chain length distribution (CLD), i.e., the number or mass fraction of macrospecies with a given number of monomers incorporated (*x* values in the present work) and its corresponding averages (e.g., the number/mass average chain length *x_n_*_/*m*_). Important dependencies here are (i) the functionality degree of the monomers, i.e., the so-called *f* values representing the maximum number of functional groups (FGs) that participate in the polymerization per original monomer (e.g., *f*_1_ = *f*_2_ = 4 in Figure 1b), and (ii) the stoichiometry, i.e., the so-called *r* value reflecting the relative presence of certain FGs at the start. In the present work, *r* = *N_A_*_,0_/*N_B_*_,0_ with *N_A_*_,0_ and *N_B_*_,0_ as the initial number of A and B FGs, considering *r* ≤ 1 making A the limiting FG. Due to the nature of the step-growth polymerization mechanism with a gradual formation of dimers (*x* = 2), trimers (*x* = 3) and ultimately *x*-mers with *x* >> 1, a high extent of reaction and thus FG conversion (>>0.95) is required to achieve high average chain lengths [1,14]. Impurities and molar imbalances, which are often aggravated by different volatility tendencies, have a deleterious effect on the quality of the final polymer product. High average chain lengths can only be obtained under ideal polymerization conditions (*r* = 1) and, in practice, can require a series of reactors, with the last frequently being a so-called solid-state reactor, operated at a lower temperature to minimize polymer degradation, but still at a sufficiently high reaction temperature to allow for sufficient FG mobility [15,16,17]. For CLD determination, experimental limitations exist, as it is non-trivial to obtain absolute CLDs and to perform reliable and time-efficient analysis, specifically for highly branched network systems. Therefore, the step-growth polymer community also employs theory to understand the success of a synthesis recipe. Originally emphasis has been on linear step-growth polymerization with AB monomers inherently minimizing the impact of a stoichiometric imbalance. However, commercially, the use of AA and BB monomers is more cost-effective, explaining why one also considers, in theoretical developments, *r* values below 1. In most theoretical work, intramolecular reactions are ignored (so no “intra”, as specified in Figure 1), and the focus is on molecular properties as a function of functional group conversion and not the reaction time.

To describe the behavior of linear step-growth polymerization systems, in 1930, Carothers proposed a pioneering equation, later denoted as the Carothers equation [18]. This equation is based on general stochastic insights and gives the degree of polymerization or number average chain length, i.e., *x_n_*, for a given (A or equivalently B) FG conversion *p* with *r* = 1 ignoring intramolecular reactions:

(1) $$x_{n}=\frac{1}{1-p}$$

This equation reaches an asymptotic value at *p* = p* = 1 and requires, in practice, a final evaluation at *p* = 0.99. The monomers AA and BB (or AB) are considered having a chain length of 1, and it is assumed that potential byproduct is removed from the reaction mixture so that equilibrium settings can be ignored (so-called kinetically controlled step-growth polymerization) [19]. Similar expressions are available to describe *x_m_* and the dispersity (*Đ*; ratio of *x_m_* and *x_n_*) as a function of *p*:

(2) $$x_{m}=\frac{1+p}{1-p}$$

(3) Đ=1+p

Flory [20,21,22] collaborated, later on, to calculate the associated mass CLD by a statistical approach based on equal reactivity of FGs:

(4) $$m_{x}=x{\left(1-p\right)}^{2}p^{x-1}$$

The extension of the Carothers equation (Equation (1)) to account for a stoichiometric imbalance (*r* < 1) with *p_A_* the FG conversion of the limiting FG A has been reported as:

(5) $$x_{n}=\frac{1+r}{1+r-2rp_{A}}$$

and has been confirmed (as well as the previous equations) by kinetic (modeling) approaches, in which time dependencies are accounted for by integrating continuity (or moment) equations or sampling reaction probabilities based on concentration variations, e.g., kinetic Monte Carlo simulations [23,24,25,26].

For branched step-growth polymerization systems, Carothers [18] modified his original equation stating that *x_n_* is dependent on the average functionality per monomer *f_av_* (Equation (6); $f_{av}=\frac{N_{mon,A,0}f_{A}+N_{mon,B,0}f_{B}}{N_{mon,A,0}+N_{mon,B,0}}$ with *N_mon_*_,*A*,0_ and *N_mon_*_,*B*,0_ being the initial number of monomer molecules based on FG A and B, respectively, and *f_A_* and *f_B_* the related functionality degrees, respectively). The equation is limited to stoichiometrically balanced reactions (*r* = 1), describing the synthesis up to the asymptotic value located at p* = 2/*f_av_*, which is denoted as the critical degree of FG conversion.

(6) $$x_{n}=\frac{2}{2-pf_{av}}$$

It can be expected that this asymptotic value (2/*f_av_*) is only a rough estimate to describe gelation. In this respect, Flory [20,21,22] and Stockmayer [27,28,29] derived an equation to predict the gel point, i.e., the point at which a polymer network is formed, resulting in an abrupt change in viscosity, which is theoretically associated with the limit of one network molecule. This was specifically done for systems consisting of the following three types of monomers: branched A-based monomer with *f* (=*f*_1_) > 2, linear BB monomer (*f*_2_ = 2), and linear AA monomer (*f*_3_ = 2), by introducing the discriminator α_c_, called the critical coefficient of branching:

(7) $$\alpha_{c}=\frac{1}{f-1}$$

The Flory–Stockmayer theory [20,21,22,27,28,29], which assumes only intermolecular reactions and equal reactivities for FGs, as in the derivation of Equations (1)–(6), states that gelation occurs if α > α_c_ as a function of *p_A_*:

(8) $$\alpha =\frac{rp_{A}^{2}\rho }{1-rp_{A}^{2}\left(1-\rho \right)}$$

with *ρ* being the initial ratio of the number of *A* FGs in the branched monomers to the total number of *A* FGs and *α* the coefficient of branching, which is defined by the probability that a certain branched unit will be joined to a second branched unit rather than to a terminal group. As derived by Flory [22], the associated *x_n_* follows from:

(9) $$x_{n}=\frac{f\left(1-\rho +\frac{1}{r}\right)+2\rho }{f\left(1-\rho +\frac{1}{r}-2p_{A}\right)+2\rho }$$

Equation (9) results in an asymptote if $p_{A}^{*}=\frac{\rho }{f}+\frac{1}{2r}+\frac{1-\rho }{2}$ representing the maximal A FG conversion that can be obtained without ring (or loop) formation due to intramolecular reactions. This is consistent with earlier findings by Carothers [18], who pointed out that if one intermolecular linkage is formed per initial monomer, all the monomers must be bound into one large molecule so that no further intermolecular reactions are possible. In this way, no full FG conversion can be reached, with for instance in the stoichiometric reaction of a bifunctional and a trifunctional monomer (*f*_1_ = *f* = 3; *f*_2_ = 2; ρ = 1; *r* = 1), p_A_* according to Equation (9) being 83%, which is 5/6 *100 as six potential AB linkages exist for five monomers (3 B_2_ and 2 A_3_). Furthermore, Stockmayer derived the corresponding equation for *x_m_* assuming *ρ =* 1, again highlighting the concept of asymptotic values:

(10) $$x_{m}=\frac{\frac{2r}{f}\left(1+rp_{A}^{2}\right)+\left(1+\left(f-1\right)rp_{A}^{2}\right)+4rp_{A}}{\left(\frac{2r}{f}+1\right)\left(1-r\left(f-1\right)p_{A}^{2}\right)}$$

Stockmayer [29] also developed an equation for the distribution representing the variation in the number fraction of molecules with *y f*-functional monomers and *z* bifunctional monomers incorporated (*r* = 1 and ρ = 1):

(11) $$f_{n;y,z}=f{\left(\frac{N_{A,0}}{N_{A,0}+N_{B,0}}\right)}^{y-1}{\left(\frac{N_{B,0}}{N_{A,0}+N_{B,0}}\right)}^{z}p_{A}^{y+z-1}{\left(1-p_{A}\right)}^{fy-2y+2}\frac{\left(fy-y+z\right)!}{y!z!\left(fy-2y+2\right)!}$$

Flory [22] and Stockmayer [27,28,29] studied well-defined step-growth polymerizations by determining the probabilities of finding various branched molecular structures in the reaction system at given FG conversions using combinatorial arguments. Starting with the assumptions of equal reactivity of FGs and no intramolecular reactions, they used probability distributions to derive expressions for the CLD and to compute *x_n_* and *x_m_* up to the theoretical limit of one molecule. For more complex systems, as encountered in industrial formulations (e.g., AB_f_ monomers), complex combinatorial analysis is required. Gordon [30] therefore adapted Good’s theory of stochastic branching, i.e., the cascade theory [31,32], in which the connectivity of chemical structures in network molecules is visually represented by rooted trees. The generation of the trees at a given FG conversion occurs by means of probability generating functions:

(12) $$F\left(\theta \right)=\underset{y}{\sum }p_{y}\theta^{y}$$

with *p_y_* denoting the probability of event *y* and *θ* a dummy variable. These functions generate all possible treelike molecules with proper weighing compatible with random combinations of reacted FGs. As the molecules are still generated from FGs at any extent of reaction, no information is stored on sequence order in the generated structures. Nevertheless, chain length averages can still be determined without first determining the CLD, and more recent results have been reported by Beginn et al. [33] and Cheng et al. [34,35]. Hillegers et al. [36] used, in turn, this approach to calculate path length—the number of chemical bonds in the path connecting two monomeric units in a molecule—distributions in an A_1_ + A_2_ + A_3_ type polymerization. However, the technique based on Equation (12) involves abstract mathematics and, as such, is difficult to use. That is why Macosko and Miller [37,38] developed a new method for calculating averages before and beyond the gel point, starting from elementary probability theory and utilizing the recursive nature of the branching process and conditional expectations. Their technique is mathematically easier to implement and yields results that are identical to those obtained by Gordon but still considers the initial assumptions of Flory and Stockmayer with no tracking of the reaction event history.

More promising is the so-called rate theory, as proposed by Stanford and Stepto [39] for linear step-growth polymerizations, in which concentration and FG dependencies are mapped as a function of reaction time and the FG conversion is calculated in parallel. This method is based on the principle that species with size *i* and with size *j* merge to form species with size *i* + *j*. In this way, the evolution of species can be described by a set of differential equations. For example, Ziff [40] confirmed that the distribution obtained by the Stockmayer theory (Equation (11)) is the solution of the corresponding (dynamic) differential equations. Furthermore, the rate theory has been used to predict CLDs and the onset of gelation both for batch reactors [41,42,43,44] and for perfectly mixed continuous flow stirred tank reactors [45,46]. More recently, the rate theory was applied by other groups as well [47,48,49,50,51,52,53,54]. Finally, Schamboeck et al. [55] applied the theory of percolation [56] on a directed random graph to derive analytical expressions describing the molecular structure of branched polymers synthesized via irreversible step-growth polymerization.

**Figure 2:**
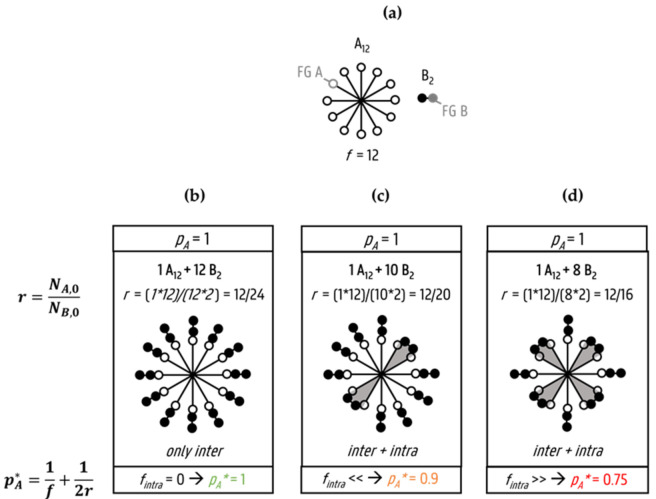
Comparison between real maximal limiting A functional group (FG) conversion (*p_A_*) being always 1 (top of each box), and as calculated by Equation (9) with *ρ* = 1 (bottom of each box; $p_{A}^{*}$; no intramolecular reactions allowed), reacting a multifunctional monomer (*f*_1_ = *f* = 12) and a bifunctional monomer (*f*_2_ = 2) as depicted in (**a**) for three (cumulative) fractions of intramolecular reactions (*f_intra_*): *f_intra_* = 0 (**b**), *f_intra_* << (**c**) and *f_intra_* >> (**d**). Equation (9) fails if intramolecular reactions are present as in (**c**,**d**) (cases with *f_intra_* > 0); rings and thus loops resulting from intramolecular reactions are highlighted in grey and are for illustration purposes formed based on short FG distances.

The main shortcoming of Equation (9) (and thus also Equations (1), (5) and (10)), and most more recently developed theories, as described above, is the neglecting of intramolecular or cyclization reactions (ring formation). It is clear from Figure 2 that Equation (9) does not hold if intramolecular reactions occur. A highly functional monomer (A_12_; *f*_1_ = *f* = 12, *ρ* = 1; Figure 2a) is depicted as fully reactive and focus is on linkage with a bifunctional monomer (B_2_; *f*_2_ = 2; Figure 2a), assuming no mobility restrictions as facilitated by selecting FGs for intramolecular reactions that are at short distance. For three values of *r* (Figure 2b–d), it is demonstrated that the predicted p_A_* according to Equation (9) is only representative if the (cumulative) fraction of intramolecular reactions *f_intra_*, which is defined as the ratio of the number of intramolecular reactions to the total number of reactions or AB-linkages formed, is zero. With a non-zero *f_intra_,* the real FG conversion of A (*p_A_*) can become 1, while Equation (9) contradicts this specifically if one assumes a high *f_intra_*, as in Figure 2d (p_A_* = 0.75 whereas *p_A_* = 1).

In linear step-growth polymerizations, intramolecular reactions are rarely considered in modeling studies, as they are rather unlikely to happen, although small rings have been mentioned [57,58,59,60], causing deviations from Equation (5) (and Equation (1)). The same holds for free radical polymerization (FRP) systems, where the occurrence of cyclization reactions has been confirmed for a limited number of cases, including, for instance, short-chain biradicals [61,62]. The situation is different if one uses multifunctional instead of bifunctional monomers in step-growth polymerization, where it is expected that intramolecular reactions play a more important role, as many FG combinations for intramolecular reactions can be identified, especially close to the “gel point”. Experimentally, the determination of this point has been investigated thoroughly. Specifically, Stafford [41] has made a summary of experimentally determined gel points, comparing them to typical theoretical values (e.g., based on Equations (8) and (9)). The deviation between experimental results and theoretical predictions has been attributed to the occurrence of intramolecular reactions. Several experimental investigations have revealed that intramolecular reactions can indeed not be neglected during network formation [63,64,65,66,67,68]. It has been further shown that the macroscopic properties of network materials are affected by these reactions [64,65].

It is thus clear that models to describe step-growth polymerization can be improved by including the occurrence of intramolecular reactions. Unfortunately, only very few attempts to treat the challenge of mapping competitive inter- and intramolecular reactions have been reported, with most of them based on the cascade theory and often focusing on the determination of the critical gelation conversion [69,70]. For instance, Jacobsen and Stockmayer [71] put forward the probability of ring formation in the step-growth polymerization of a bifunctional monomer using Gaussian conformational statistics for the growing chain. The probability that a chain is in a ring formation is claimed to be proportional to *n_l_*^−3/2^ with *n_l_* the number of links. The ring-chain equilibria are obtained by comparing stochastic variations of meeting ends of different chains and of the same chain in a small volume element. Furthermore, Gordon et al. [69] and Dusek et al. [70] combined the cascade theory with Gaussian statistics to include the probability of ring formation. This approach has been later discussed by Kricheldorf et al. [59], who were able to identify cyclic products by means of matrix-assisted laser desorption/ionization-time of flight (MALDI-TOF) mass spectrometry.

The deterministic kinetic approach has also been extended to account for intramolecular reactions [72,73,74,75,76,77,78,79,80], but is typically limited to very low FG conversions due to the complexity of integrating the associated differential equations. A comparison with the cascade theory revealed that the simulated impact of intramolecular reactions is lower, as also shown by Mikes and Dusek [81], using in silico generation of polymer chains. In the work of Kumar et al. [73,74], next to the gel point, the CLD has been calculated in the presence of intramolecular reactions for the polymerization of A_f_-type monomers with themselves. In their kinetic model based on integrating continuity equations, they defined *P_p_*_,x_ as the species having chain length *x* with *p* intramolecular bonds, and the rate coefficient for the intramolecular reaction was determined using the percolation theory [56]. Another approach relates to lattice-based simulations, in which FGs are placed at fixed positions to mimic the structure of the network molecules [82,83,84]. For instance, Cameron et al. [83,84] developed a computer-based lattice model for the step-growth polymerization of an AB_2_ monomer that allows the simultaneous and explicit occurrence of inter- and intramolecular reactions of A and B FGs according to stochastic selections of pairs adjacent on the lattice. However, conventional lattice-based models are treating time dependencies in a simplified manner. They are preferably extended considering kinetic rules such as varying reaction probabilities, as recently done in the field of polymer brush synthesis [85,86].

Despite the progress made in the field, kinetic modeling tools for step-growth network synthesis, with a limited number of assumptions and focusing both on FG conversion and time dependencies, remain scarce. In such studies, one strives not only for a differentiation between inter- and intramolecular reaction rates but also for automated validation if network synthesis reactions can take place upon a random selection of FGs. For example, FGs belonging to separate molecules that are incapable to diffuse will not react, but in a model with constant intermolecular reactivities and no time-dependent tracking of connectivities, as is commonly the case, this physical constraint will be ignored. Similarly, intramolecular reactions can be expected not to go on forever with the same reactivity, opposed to what is assumed in most more advanced models. Polymerization media are viscous, certainly for network systems at high FG yield. Variable, thus apparent, inter- and intramolecular rate coefficients, are therefore desired in model implementations [87,88,89,90,91].

With the recent advent of matrix-based kinetic Monte Carlo (*k*MC) simulations, it is possible to explicitly track the connectivities of (co)monomer units [92,93,94,95], therefore allowing the calculation of time-dependent CLDs, differentiating between the contribution of linear and branched/crosslinked species, and acknowledging the apparent nature of rate coefficients. Such high-level modeling platforms facilitate reverse engineering, in which the model output can be compared with simplified but pragmatic theories that consist only of a limited number of equations. In the present work, we demonstrate that matrix-based *k*MC simulations allow the grasping of the interplay between inter- and intramolecular reactions at any time and FG conversion, considering theoretical cases of constant and varying reactivities. The main focus is on the reaction between A_f_ and B_2_, but examples for other *f* combinations are also considered. It is shown that the use of asymptotes from commonly applied theories is biased if intramolecular reactions matter. This bias is even stronger if mobility restrictions, and thus, diffusional limitations are relevant. Reverse engineering is also applied to test hypotheses regarding the future development of advanced (pseudo-)analytical approaches. Guidelines are additionally provided for the experimentalist to verify the actual value for the ratio of the rate coefficient for intramolecular reactions, compared to that for intermolecular reactions. The present work, therefore, contributes to a dedicated kinetic understanding of step-growth polymer synthesis up to very high FG conversions.

## 2. Kinetic Monte Carlo Modeling Details

**Figure 3:**
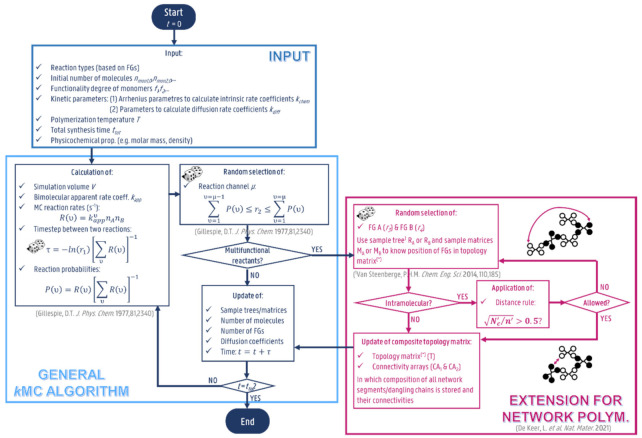
Flowsheet of kinetic Monte Carlo (*k*MC) model to follow the kinetics during network synthesis on the level of the individual segment/molecule. In purple, the specific steps related to the selection of FGs for an inter- and intramolecular reaction in polymer network synthesis. For comparison with simplified models, one can skip the calculation of apparent intermolecular reactivities (*k_app_* values) and the distance rule for intramolecular reactions. FG: functional group; *n*’/*N_c_*’: amount of monomer units/crosslinking points between the selected FGs; *n*_X_: number of X type molecules; R/P: MC reaction rate/probability; *r_i_*: random number; *t*_(*tot*)_: (total) time; *ν*: MC reaction channel. Figure S3 in the Supplementary Materials gives extra information on the selection of the specific FGs in a molecule.

The main steps of the matrix-based *k*MC model to study the kinetics of step-growth polymer network synthesis are given in Figure 3 and build on the principles as outlined in previous work [92,96,97,98,99,100,101], with the core being the storage of individual reaction events and the associated compositions molecule by molecule. The reactions are defined based on the FGs that need to chemically rearrange, but of course, need to be linked to specific molecules due to the multifunctional nature of network synthesis. The steps that are related to the differences in the treatment of the selection of FGs to execute inter- and intramolecular reactions, as important for this work, are highlighted in purple. The source code was written in Fortran programming language (IBN, New York, NY, USA) and compiled using an Intel Fortran Compiler. 

As can be seen in Figure 3, the *k*MC model requires as input: (i) a list of all reaction types and their corresponding chemical and diffusion parameters (including the polymerization temperature), (ii) the initial number of all types of molecules with the possibility to select monomers with different functionality degrees (e.g., *f*_1_ = 3, *f*_2_ = 4; …), (iii) the total synthesis time (*t_tot_*) and (iv) properties of all types of molecules (e.g., molar masses and densities). In the present work, the volume is fixed at 1.0 × 10^−15^ L, checking numerical convergence for the associated initial number of molecules, as shown in Figure S1 of the Supplementary Materials as a function of *p_A_*, and Figure S2 of the Supplementary Materials as a function of time for a reference condition. Subsequently, for every reaction type, the corresponding MC rate (s^−1^) is calculated based on the reaction possibilities and the conventional rate coefficient, correcting with the simulation volume and the Avogadro number for intermolecular reactions and without such correction for intramolecular reactions. For intermolecular reactions, apparent rate coefficients are generally used, as calculated using so-called encounter pair models [90,102,103], to account for viscosity effects and dependencies on the number of monomer units. To allow for comparison with models with constant (intrinsic) intermolecular reactivities (e.g., the analytical approach of Flory), such a switch from intrinsic to apparent rate coefficients can be omitted in the algorithm (*k_inter,intr_* is formally taken as 1 mol L^−1^ s^−1^ in this work). The time step between randomly selected reaction events is then calculated using a first random number *r*_1_ and normalization with the total MC reaction rate. In the next step, the selection of the reaction type to be executed (e.g., an intermolecular reaction between the specific FG A and B) is performed using a second random number *r*_2_, favoring reaction types with a higher probability in line with the original algorithm for elemental and thus non-distributed systems as pioneered by Gillespie [104].

If FGs are involved for the reaction type selected, which are present multiple times in the same molecule, one needs to ideally know the structure of all the molecules in the reaction mixture. In the present work, information about the composition of all individual network segments and dangling chains (thus free FGs) and their connectivities are stored in an additionally included so-called composite topology matrix (see Figure S3 in the Supplementary Materials), consisting of the core topology matrix (rows: compositions of segments) and two additional connectivity arrays (rows: information to which connectivity points segments belong). Extra sampling matrices (see Figure S3 in the Supplementary Materials) are used for each type of FG to store the position of unreacted FGs of that type in the topology matrix. For the selection of the FGs involved in the selected reaction, binary sampling trees are used [97] (see Figure S3 in the Supplementary Materials). A binary sampling tree is created for every type of FG storing molecular information, in which the tree leaf nodes represent the number of FGs of that type in a certain molecule. By randomly selecting a FG, a certain network molecule is selected. The position of the FG in the topology matrix is found using a so-called sampling matrix. In this way, the topology matrix and the connectivities can be properly updated. Similarly, this is done for a possible second reaction partner. Here, for intermolecular reactions, it is checked if the FGs belong to two different molecules, and for intramolecular reactions, it is analogously verified if the sampled FGs belong to the same molecule. For the intramolecular reactions, mobility restrictions are also accounted for by a distance rule based on the local root mean squared end-to-end distance [105]. This rule verifies if the FGs selected for the intramolecular reaction are close enough to each other based on a calculation of the compactness of the local region of the FGs, as accessible from the molecular information in the composite topology matrix. Only if the compactness is high enough with respect to a tunable system-dependent target value, the intramolecular reaction is allowed. As the current work is of a purely illustrative nature, a default value of 0.5 is considered. For comparison with simplified (kinetic) models, in which constant intramolecular reactivities are assumed, the application of this distance rule can be skipped, and thus, the algorithm simplified.

In the last step, all related sample trees, sample matrices, number of molecules, diffusion coefficients and the reaction time are updated. If the final synthesis time (or a certain FG conversion) is reached, the algorithm iteration is closed. If not, the algorithm continues so that the next reaction event can occur at the next selected time. At any synthesis time (or FG conversion), the desired molecular characteristics can be plotted. In the present work, the emphasis is only on basic molecular properties as defined in the introduction, such as *x_n_*, x*_m_*, *Đ* and the number/mass CLD.

## 3. Results and Discussion

### 3.1. From Flory(-Stockmayer) Analytical Equations to kMC Prediction of the “Inter-Intra Competition”

**Figure 4:**
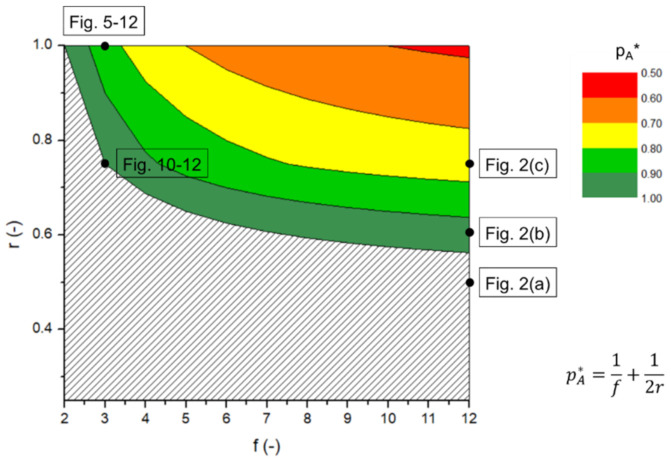
Analytically obtained contour plot for the effect of *r* (*r* = *N_A_*_,0_/*N_B_*_,0_ with *N_A_*_,0_ and *N_B_*_,0_ the initial number of A and B functional groups (FGs)) and *f* (functionality degree of multifunctional monomer; A_f_; other monomer is bifunctional; B_2_) on the limiting FG conversion of A ($p_{A}^{*}$) using the equation derived by Flory [22] (*ρ* = 1 in Equation (9); no consideration of intramolecular reactions); in the shaded area the equation is not applicable as no asymptote exists. The specific (*f*,*r*) combinations considered in other figures are also indicated.

For step-growth network synthesis with A_f_ (*f* > 2) and B_2_ (ρ = 1), Equation (9), which reflects the variation of *x_n_* as a function of *p_A_* without intramolecular reactions and diffusional limitations, thereby considering only (constant) intermolecular reactivities, results in a vertical asymptote located at $p_{A}^{*}=\frac{1}{f}+\frac{1}{2r}$. This value represents the maximal A FG conversion that can be obtained without the presence of ring formation due to intramolecular reactions. In Figure 4, the effect of *f* and *r* on p_A_* is shown by a contour plot. It can be seen that very-high to complete A FG conversion can be reached for all *f* values if *r* is sufficiently low (dark green area). Mathematically speaking, values above one can even be obtained based on Equation (9), indicating the physical absence of an asymptote (shaded area below the dark green area). The lowest p_A_* values (red area) are obtained for high *f* values under initial stoichiometric conditions (*r* = 1). Here, a limited number of intermolecular linkages (without the possibility of intramolecular reactions) already brings in the limit of one large network molecule, inducing many free A FGs in the final structure (i.e., a low p_A_*). This is, for instance, the case in Figure 2d, as also highlighted by a box in Figure 4.

**Figure 5:**
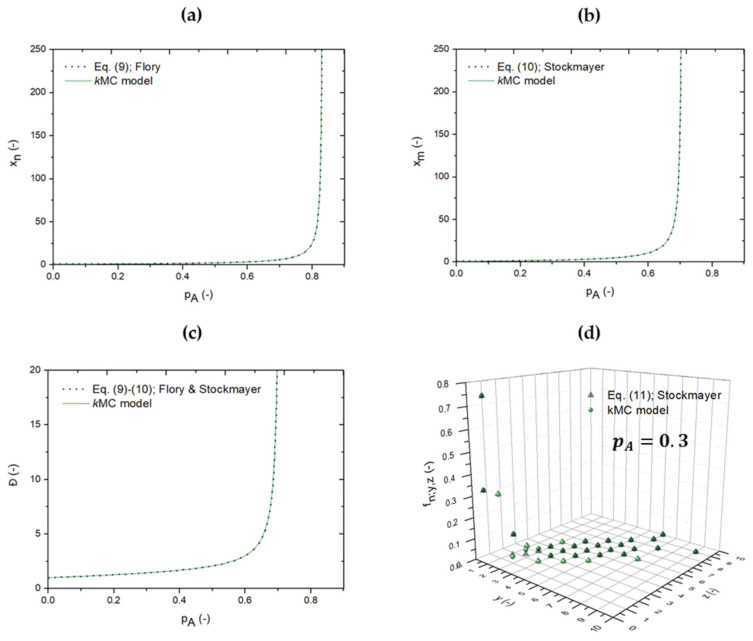
Benchmark between equations derived by Flory [22] and/or Stockmayer [27,28] (black) and the kinetic Monte Carlo (*k*MC) model in the present work without intramolecular reactions and diffusional limitations for network synthesis, and thus, constant intermolecular reactivities (green), starting from a multifunctional (*f* = 3; A based; A_3_) and bifunctional monomer (B_2_) and stoichiometry of A and B functional groups (FGs; *r* = 1): (**a**) number average chain length *x_n_* as a function of the A FG conversion *p_A_*, (**b**) mass average chain length *x_m_* as a function of *p_A_*, (**c**) dispersity *Đ* as a function of *p_A_*, and (**d**) *f_n;y,z_* distribution (*p_A_* = 0.3) with *f_n_*_;y,z_ the number fraction of molecules with *y f*-functional monomers and *z* bifunctional monomers incorporated. Other *p_A_*: Figure S4 in the Supplementary Materials.

As indicated above, the *k*MC model considered in this work (Figure 3) enables to account for both inter- and intramolecular reactions, as well as diffusional limitations or equivalently mobility constraints. A natural first step is to validate if, under simplified model assumptions, the detailed *k*MC model is consistent with earlier developments in the theoretical field. The focus here is on network synthesis with a 3-functional monomer (A_3_) and a bifunctional monomer B_2_. In Figure 5a,b, it is shown that the developed *k*MC model benchmarks with the formulas for *x_n_* and *x_m_* developed by Flory [22] (Equation (9)) and Stockmayer [27,28] (Equation (10)) under the assumption that no intramolecular reactions take place, diffusional limitations are absent, and stoichiometry of A and B FGs holds (*r* = 1 and ρ = 1). For both *x_n_* (Figure 5a) and *x_m_* (Figure 5b), an excellent match between the black dashed (analytical) and green (*k*MC) solutions can be witnessed, highlighting the accuracy of the stochastic modeling framework. For completeness, the dispersity (*Đ*) variation is also shown in Figure 5c, and as both *x_n_* and *x_m_* variations match, it is not surprising that there is also a match observed between the black dashed and green solid line. Notably, at high *p_A_*, *Đ* values much larger than 2, as maximally accessible based on linear step-growth polymerizations, result (Equation (3)), indicative of a larger molecular heterogeneity in step-growth network synthesis.

Furthermore, the number distribution for *p_A_* = 0.3, according to Stockmayer [29], is shown in Figure 5d as black symbols (Equation (11)) and is compared with the *k*MC prediction (green symbols). Results at other FG conversions are shown in Figure S4 in the Supplementary Materials. It follows that both calculation methods are in agreement, but some slight deviations can be observed, which can be associated with the numerical evaluation of Equation (11) involving large powers and the factorial function of large integer values. Preference should therefore be given to the *k*MC outcome as it inherently tracks all y-z combinations discretely.

**Figure 6:**
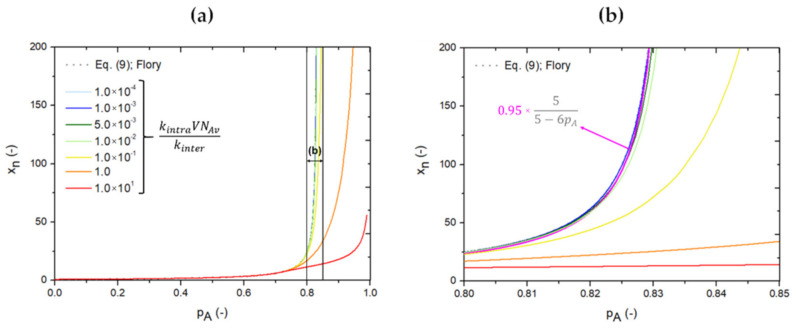
Going beyond the Flory equation [22] for step-growth network synthesis with a multifunctional monomer A_3_ and a bifunctional monomer B_2_ by including intramolecular reactions in the kinetic Monte Carlo (*k*MC) simulations, for the case of stoichiometry in A and B functional groups (FGs; *r* = 1) and without mobility restrictions or diffusional limitations, and thus considering constant reactivities. This is done by evaluating the effect of $\frac{k_{intra}VN_{av}}{k_{inter}}$ on the number average chain length *x_n_* as a function of the A FG conversion (*p_A_*); the Flory equation [22] (Equation (9); *ρ* = 1) is depicted as a grey dashed line; subplot (**b**) is a zoom of (**a**) for a small *p_A_* range with a 5% deviation from the equation by Flory, additionally indicated by a fuchsia dashed line; *k_intra/inter_*: intra/intermolecular rate coefficient; *V*: simulation volume; *N_Av_*: Avogadro number; *k*MC simulations based on Figure 3.

With a successful benchmark under idealized conditions in Figure 5, one can now gradually increase the complexity of the network synthesis *k*MC modeling to mimic the real situation more. In the first phase, emphasis is on accounting for the competition of inter- and intramolecular reactions, still ignoring mobility restrictions and, thus, diffusional limitations. In other words, for both types of reactions, constant reactivities are still considered during the *k*MC simulations. In Figure 6, it is shown how the *x_n_* profile predicted by Flory [22] (Equation (9); *ρ* = 1) is altered if intramolecular reactions are accounted for, still considering the reaction of A_3_ and B_2_ (*f =* 3*, r =* 1 and *ρ =* 1). This is done by considering different values of the dimensionless parameter $\frac{k_{intra}VN_{Av}}{k_{inter}}$, with *k_inter/intra_* the intermolecular/intramolecular rate coefficient, *V* the simulation volume and *N_Av_* the Avogadro number. A variation of $\frac{k_{intra}VN_{Av}}{k_{inter}}$ between 1.0 × 10^−4^ and 1.0 × 10^1^ is included, thus addressing both cases of low and high relative importance of intra- versus intermolecular reactions. Figure 6a highlights the invalidity of the Flory equation [22] for most $\frac{k_{intra}VN_{Av}}{k_{inter}}$ parameter values. In more detail, it can be seen, in the zoom of this subfigure (Figure 6b), that deviations of more than 5%, with respect to the Flory equation (fuchsia line) [22], occur already if $\frac{k_{intra}VN_{Av}}{k_{inter}}$ is higher than 1.0 × 10^−3^. Accordingly, considering sufficiently long reaction times (see Figure S5a in the Supplementary Materials), *p_A_* can be increased. In any case, for extremely long reaction times, one obtains the limit of complete A FG conversion (*p_A_* = 1), but for larger $\frac{k_{intra}VN_{Av}}{k_{inter}}$ one remains with a strong restriction in *x_n_*. A similar conclusion can be made for the *x_m_* profile starting from the Stockmayer equation [27,28] (Equation (10)), as illustrated in Figure S5c in the Supplementary Materials.

### 3.2. Reverse Engineering and Dimensionless Analysis for (Pseudo-)Analytical Descriptions

As the *k*MC results in Figure 6 with no mobility constraints highlight that deviations from conventional theory are obtained if one also considers intramolecular reactions, one can apply reverse engineering to identify advanced (pseudo-)analytical descriptions under the assumption of constant reactivities for both inter- and intramolecular reactions. A good starting point is a set of two types of differential equations regarding number variations with respect to reaction time t. To study the overall kinetics of network synthesis, one needs to know at least the variation of the number of molecules (*N*) and the variation of the number of free or unreacted FGs, either of type of A or B (*N_A_* or *N_B_*). Interestingly, *N* varies as a function of *t* due to intermolecular reactions but not due to intramolecular reactions:

(13) $$\frac{dN}{dt}=-r_{inter}\left(VN_{Av}\right)=-k_{inter}{\left(VN_{Av}\right)}^{-1}{\left(N_{A}N_{B}\right)}_{diff}$$

in which *r_inter_* represents the volumetric reaction rate (mol L^−1^ s^−1^) of intermolecular reaction and ${\left(N_{A}N_{B}\right)}_{diff}$ represents the total number of combinations of A and B FGs belonging to different molecules. This number codetermines the total number of A and B remaining FG combinations ($N_{A}N_{B}$), with the principle of conservation allowing to write at any t:

(14) $$N_{A}N_{B}={\left(N_{A}N_{B}\right)}_{diff}+{\left(N_{A}N_{B}\right)}_{same}$$

in which ${\left(N_{A}N_{B}\right)}_{same}$ represents the total number of combinations of A and B FGs belonging to the same molecules. The variation of the number of unreacted FGs A and B follows in turn by considering both ${\left(N_{A}N_{B}\right)}_{diff}$ and ${\left(N_{A}N_{B}\right)}_{same}$:

(15) $$\;\;\;\frac{dN_{A}}{dt}=\frac{dN_{B}}{dt}=-\left(r_{inter}+r_{intra}\right)\left(VN_{Av}\right)=-k_{inter}{\left(VN_{Av}\right)}^{-1}{\left(N_{A}N_{B}\right)}_{diff}-k_{intra}{\left(N_{A}N_{B}\right)}_{same}$$

in which *r_intra_* represents the volumetric reaction rate (mol L^−1^ s^−1^) of intramolecular reaction. To integrate Equations (13) and (15), an explicit expression for ${\left(N_{A}N_{B}\right)}_{diff}$ and ${\left(N_{A}N_{B}\right)}_{same}$ is needed, which from an analytical point of view requires simplifications. It can, for example, be assumed that all molecules have the same number of unreacted A FGs and the same number of unreacted B FGs so that every molecule behaves as an average molecule. With such averaging, as typical in the field of the method of moments [106,107], each molecule has $\frac{N_{A}}{N}$ FGs A and $\frac{N_{B}}{N}$ FGs B so that the following expressions are obtained:

(16) $${\left(N_{A}N_{B}\right)}_{same}=\frac{N_{A}}{N}\frac{N_{B}}{N}N=\frac{N_{A}N_{B}}{N}$$

(17) $${\left(N_{A}N_{B}\right)}_{diff}=N_{A}N_{B}-\frac{N_{A}N_{B}}{N}$$

Substitution of these equations in Equations (13) and (15) leads to the following differential equations with only *N*, *N_A_* and *N_B_* varying with *t*:

(18) $$\frac{dN}{dt}=-k_{inter}{\left(VN_{Av}\right)}^{-1}\left[\left(\frac{N-1}{N}\right)N_{A}N_{B}\right]$$

(19) $$\;\;\;\;\;\;\frac{dN_{A}}{dt}=\frac{dN_{B}}{dt}=-k_{inter}{\left(VN_{Av}\right)}^{-1}\left[\left(\frac{N-1}{N}\right)N_{A}N_{B}\right]-k_{intra}\left(\frac{1}{N}\right)N_{A}N_{B}$$

If *k_intra_* = 0 s^−1^, the (instantaneous) depletion of the number of molecules and A/B FGs is identical, as in this case for each intermolecular reaction between A and B, the number of molecules decreases by one, as was also derived by Flory [22]. If one molecule is left (*N* = 1), only the second term in Equation (19) remains and only intramolecular reactions can take place. At the start, the opposite situation exists with almost exclusively intermolecular reactions taking place (1/*N* -> 0; (*N* − 1)/*N* -> 1). In between, at most *t* values, one thus observes a competition between inter- and intramolecular reactions. Note that to obtain the conventional volumetric reaction rate *r_tot_* (= *r_inter_* + *r_intra_*) in mol L^−1^ s^−1^, one needs divisions by the product *VN_Av_* on the left and right hand side in Equations (18) and (19).

**Figure 7:**
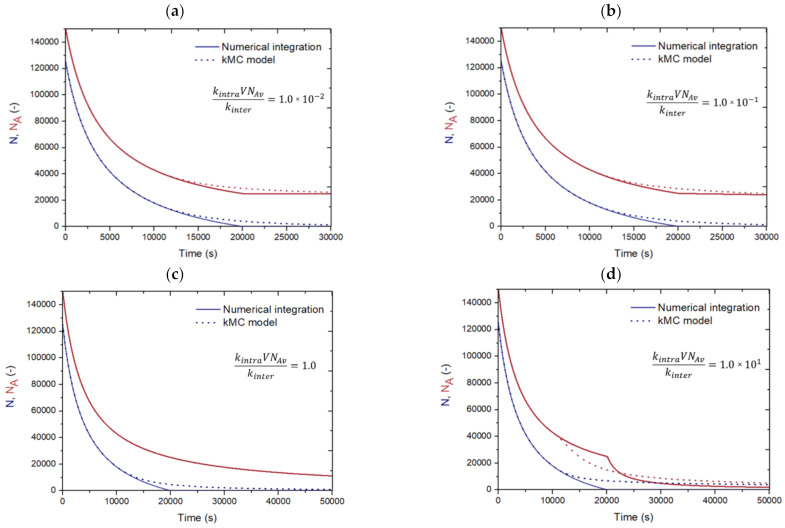
Comparison between simplified analytical descriptions in the presence of intramolecular reactions assuming that unreacted FGs A and B are evenly distributed over the molecules (solid lines; integration of Equations (18) and (19) with Maple) and the solution obtained from *k*MC simulations (dotted lines, Figure 3) for the number of molecules *N* (blue) and the number of unreacted FGs A (red) as a function of time (s) for different values of $\frac{k_{intra}VN_{Av}}{k_{inter}}$: (**a**) $\frac{k_{intra}VN_{Av}}{k_{inter}}=1.0\times 10^{-2}$, (**b**) $\frac{k_{intra}VN_{Av}}{k_{inter}}=1.0\times 10^{-1}$, (**c**) $\frac{k_{intra}VN_{Av}}{k_{inter}}=1.0$ and (**d**) $\frac{k_{intra}VN_{Av}}{k_{inter}}=1.0\times 10^{1}$; no mobility restrictions are accounted for; *V*: volume; *N_Av_*: Avogadro number; the assumption of average molecules is incorrect as no match is observed between the dashed and solid lines.

However, as shown in Figure 7, the assumption of the same number of *N_A_* and *N_B_* in each molecule is only representative at the lower times before intramolecular reactions start to really play a role. In this figure, for four $\frac{k_{intra}VN_{Av}}{k_{inter}}$ values, a comparison is made between the solution obtained upon numerical integration of Equations (18) and (19) (solid lines), and the solution from the *k*MC model for the network synthesis (dashed lines), still considering *f* = 3, *r* = 1 and ρ = 1 and still ignoring mobility constraints. It follows that, by numerical integration with average molecules, intramolecular reactions are only important once intermolecular reactions no longer occur, leading to incorrect second-order discontinuities. In the *k*MC simulations, while explicitly acknowledging molecular variations, intramolecular reactions start to play a role earlier. The corresponding results as a function of *p_A_* are shown in Figure S6, in the Supplementary Materials, and confirm that the deviations are manifested at the very high *p_A_* range. The deviations in Figure 7 indicate that at many *t* values, the consideration of an average number of A/B FGs per molecule is incorrect. In other words, it follows that the number of unreacted A and B FGs in the molecules, and thus ${\left(N_{A}N_{B}\right)}_{same}$, depend*s* on the size of the molecule or its number of monomer units.

**Figure 8:**
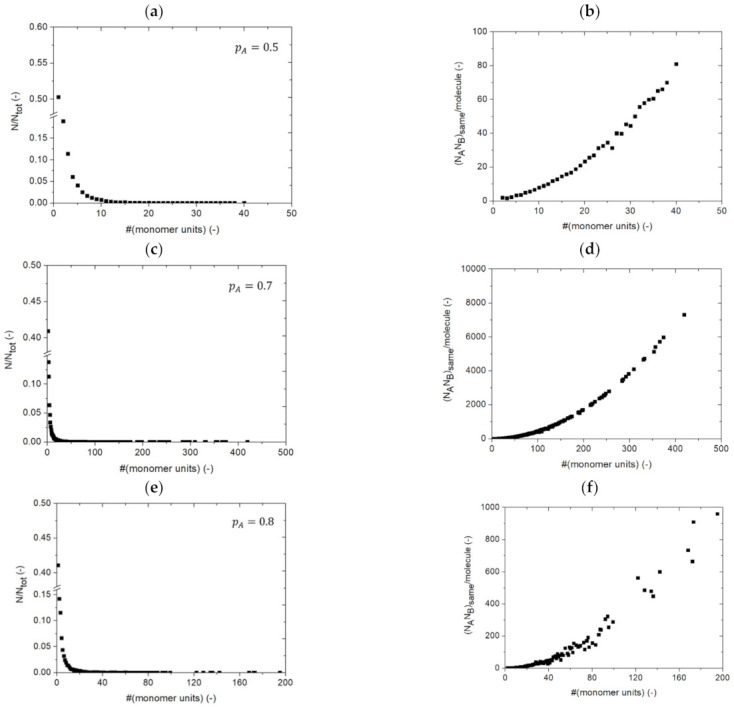

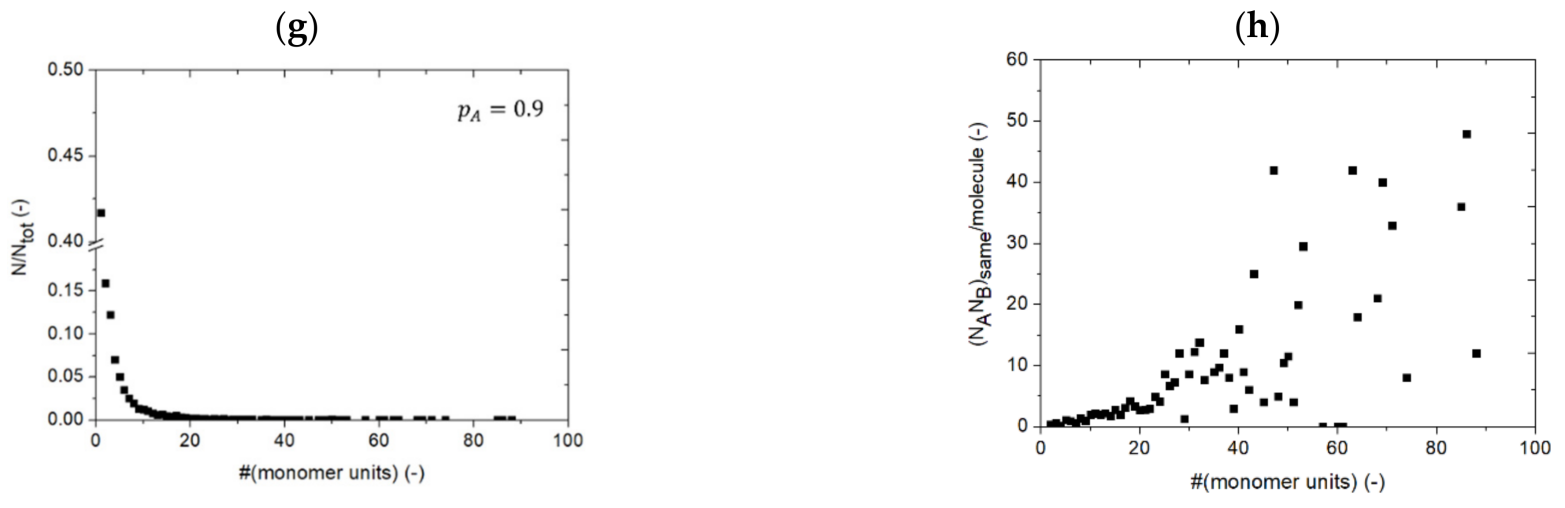
Chain length distribution (CLD; left) and the (absolute) distribution of the number of combinations of A and B functional groups (FGs) belonging to the same molecule ${\left(N_{A}N_{B}\right)}_{same}$ according to number of monomer units (right) for four A FG conversions: (**a**,**b**) *p_A_* = 0.5, (**c**,**d**) *p_A_* = 0.7, (**e**,**f**) *p_A_* = 0.8 and (**g**,**h**) *p_A_* = 0.9, considering $\frac{k_{intra}VN_{Av}}{k_{inter}}=1.0\times 10^{1}$; *k_intra/inter_*: intra/intermolecular rate coefficient; *V*: simulation volume; *N_Av_*: Avogadro number; kinetic Monte Carlo simulations based on Figure 3 (simplification with no mobility restrictions).

To see which distribution is needed to properly calculate ${\left(N_{A}N_{B}\right)}_{same}$, one can rely on the *k*MC simulations, in which the combinations per molecule size can be retrieved, as the structure of each separate molecule is stored as a function of *t*. This distribution is shown in Figure 8 (right) for four values of *p_A_* (0.5; 0.7; 0.8; 0.9) for $\frac{k_{intra}\left(VN_{Av}\right)}{k_{inter}}$ equal to 1.0 × 10^1^. For completeness, in Figure 8 (left), the corresponding number CLDs are provided, with (*N_tot_*) the total number of molecules at the given *p_A_*. At any *p_A_* an increase in ${\left(N_{A}N_{B}\right)}_{same}$ per molecule is observed with increasing molecule size. With increasing *p_A_*, the distributions on the right become more complex due to the increasing impact of intramolecular reactions. But, as can be seen, even at the lower *p_A_*, a significant variation exists, demonstrating that the assumption of an average number of FGs per molecule indeed is invalid.

At the larger *p_A_*, the distribution in Figure 8 (right) is thus less-defined, with a broader scatter in both the *x* and *y* direction so that it is not straightforward to introduce a single fitting equation type to describe the distribution at each *p_A_* and introducing it in the integration of Equations (13) and (15). This also implies that so-called conditional MC tools [108], assuming predefined distributions for sampling, are simplified and not recommended for detailed kinetic analysis. Overall, it can thus be concluded that the approach behind Equations (18) and (19) is too approximate as molecular heterogeneity matters in step-growth polymer network synthesis once the relative contribution of intramolecular reactions starts to become kinetically relevant.

An alternative approach toward a pseudo-analytical solution is to investigate the *t* dependent variation of the cumulative fraction of intramolecular reactions (*f_intra_*_(cum)_), aiming at the identification of a generic (simplified) *t* dependent formula, still considering constant inter- and intramolecular reactivities (no mobility constraints) and linking it with the *t* variation for *N*. In the derivation of Flory [22], the decrease of *N* as a function of *p_A_* is linear. However, if intramolecular reactions are occurring, this is no longer true. The decrease in *N* is then:

(20) $$N=N_{0}-\left(1-f_{intra}\right)N_{A,0}p_{A}$$

As only a fraction $1-f_{intra}$ of the reactions between the functional groups A and B contribute to the decrease in *N*. Information on the variation of the instantaneous fraction of intramolecular reactions, *f_intra,inst_*, being the time derivative of *f_intra_*, is thus required, so that solving of Equation (20) together with Equations (13) and (15), taking into account the definition of *f_intra,inst_* ($f_{intra,inst}=\frac{r_{intra}}{r_{inter}+r_{intra}}$) and *p_A_* ($p_{A}=\frac{N_{A,0}-N_{A}\;}{N_{A,0}}$), results in the *t* variation of *N* and *N_A/B_*. Bearing in mind the complex subplots in Figure 8 (right), dimensional analysis is recommended to elegantly predict $f_{intra,inst}$, with the most important theorem, the Buckingham π theorem, which can be formally stated as *“If a physically meaningful equation involving a certain number of physical variables exists, then this equation can be rewritten in terms of a set of dimensionless parameters (*$\pi_{1}$, $\pi_{2}$*,...) constructed from the original variables”*. Applied to the polymerization case at hand, the quantities $k_{intra}$ (s^−1^), $k_{inter}$ (L mol^−1^ s^−1^), *t* (s), *V* (L), *C_A_*_,0_ (mol L^−1^) and *C_B_*_,0_ (mol L^−1^) can be listed and combined at first sight, formally acknowledging the probability for inter- and intramolecular reactions, as follows:

(21) $$\pi_{1,0}=k_{intra}{\left(C_{A,0}C_{B,0}\right)}_{same}{\left(VN_{Av}\right)}^{2}t\;\;\;\;\;\;\;\;\;\;\;\;\;$$

(22) $$\pi_{2,0}=k_{inter}{\left(C_{A,0}C_{B,0}\right)}_{diff}\left(VN_{Av}\right)t\;\;\;\;\;\;\;$$

in which the subscript “0” is considered to highlight the initial attempt. As shown in Section S2 of the Supplementary Materials, relying on typical relations (e.g., $N_{A,0}=f_{A}N_{mon,A,0}$ in which *N_mon_*_,*A*,0_ represents the initial number of molecules with FG A) and using the *k*MC model for verification, these two dimensionless numbers can be further updated as follows:

(23) $$\pi_{1}=k_{intra}\left(\frac{rf_{A}f_{B}-rf_{B}-f_{A}}{f_{A}+rf_{B}}\right)\left(\frac{f_{A}f_{B}-rf_{B}-f_{A}}{f_{A}+rf_{B}}\right)N_{0}^{2}t$$

(24) $$\pi_{2}=k_{inter}\left(\frac{1}{\left(\frac{MM_{FG}}{\rho_{FG}}\right)}\right)\frac{rf_{A}f_{B}}{\left(r+1\right)\left(f_{A}+rf_{B}\right)}N_{0}t$$

in which *MM_FG_* (g mol^−1^) and *ρ_FG_* (g L^−1^) represent, respectively, the molar mass and the density of FG A and B (for simplicity these are considered equal for FG A and B, thus: *MM_FG,A_ = MM_FG,B_* = *MM_FG_* and *ρ_FG,A_* = *ρ_FG,B_* = *ρ_FG_*). To validate these updates, in Section S2 of the Supplementary Materials, it is demonstrated that with these two more advanced numbers, an increase of the rate coefficients, *k_inter/intra_*, with a certain given factor combined with a decrease of *t* with that same factor, lead to dimensionally equivalent polymerization outcomes, as expected. Other illustrations in the same section constitute the cases of imposed changes in the monomer functionalities, *f_A_* and *f_B_*, or the initial number of molecules, *N*_0_, or the stoichiometric imbalance factor, *r*. These changes are less trivial because they skew the competition between the intra- and the intermolecular reactions in a complex manner. For instance, a change from a system with *f_A_* = 12 and *f_B_* = 10 to a system with *f_A_* = 12 and *f_B_* = 12, maintaining everything else equal, promotes the intramolecular reaction by 25% ($c_{1}^{2}$ = 1.25; see Section S2 in the Supplementary Materials) and the intermolecular reaction only by 10% (*c*_2_ = 1.1; see Section S2 in the Supplementary Materials). As shown in the Supplementary Materials, dividing the rate coefficients respectively by the factors 1.25 and 1.1 leads to a dimensionally similar solution. Hence, $\pi_{1}$ and $\pi_{2}$ as defined by Equations (23) and (24) can be assumed as reliable dimensionless groups that determine the competition between inter- and intramolecular reaction for the polymerization system at hand, suggesting that a relationship for *f_intra,inst_* as a function of $\pi_{1}$ and $\pi_{2}$ should exist.

**Figure 9:**
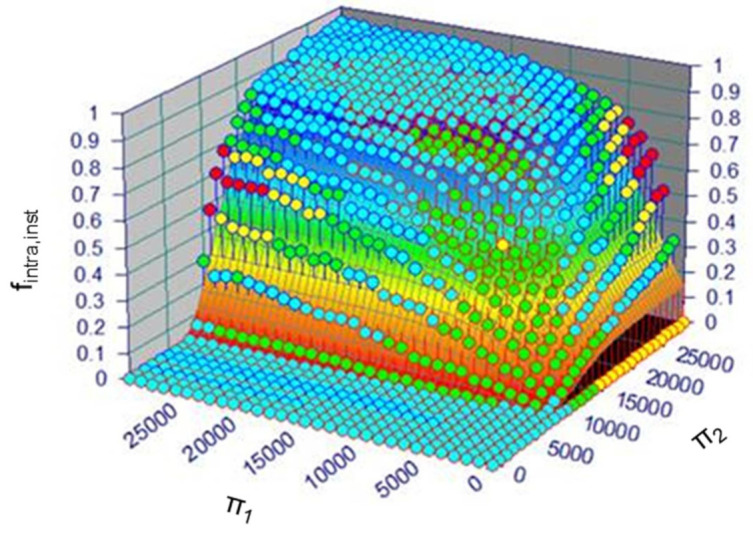
Surface fitting to represent the instantaneous fraction of intramolecular reactions, *f_intra,inst_*, as a function of two dimensionless numbers ($\pi_{1}$ and $\pi_{2}$; Equations (23) and (24)) covering the initial synthesis parameters (no mobility constraints taken into account); symbols represent kinetic Monte Carlo (*k*MC) simulation results.

Indeed, as shown in Figure 9, a surface is obtained for *f_intra,inst_* in the analytical form $\exp\left(-\exp\left(-\frac{\pi_{1}-c_{1}}{c_{2}}\right)\right)*\exp\left(-\exp\left(-\frac{\pi_{2}-c_{3}}{c_{4}}\right)\right)$ (with *c_i_*, *i* = 1…4 representing constant numbers), by fitting to the *k*MC simulation results over a broad range of input variable variations, as represented by symbols. The reported surface can be utilized to grasp the relation between initial conditions and product outcome, and one can also implement it to stepwise integrate Equations (13), (15) and (20). However, this procedure remains tedious, and a major caveat is still that the derivation of the dimensionless numbers has been performed in the absence of mobility constraints, which are highly relevant (as illustrated in the next subsection).

For completeness, variations of *f_intra,inst_* as a function of *t* are shown in Figure S7 (left) in the Supplementary Materials for several $\frac{k_{intra}VN_{Av}}{k_{inter}}$, considering the output facilities of the detailed *k*MC model but neglecting mobility constraints and thus diffusional limitations. In Figure S7 (right) in the Supplementary Materials, the corresponding variations of *N* and the raw data on MC probabilities for inter- and intramolecular reactions are provided as well. It follows that, consistent with the results in Figure 7, at the lower times, only intermolecular reactions are relevant. At those times, both the intermolecular MC rate and *N* gradually decrease up to the moment that more, large-molecules are formed, and thus more intramolecular FG combinations are possible, which is consistent with the trend in the right column of Figure 8 from top to bottom. The intramolecular MC rate then increases, and once intramolecular reactions are frequently occurring, the relevance of the intermolecular reactions becomes less. For larger $\frac{k_{intra}VN_{Av}}{k_{inter}}$ this increase in the intramolecular MC rate is more evident, explaining the more rapid increase in *f_intra,inst_*. Eventually, also the intramolecular MC rate decreases, and the system starts to converge to the allowed limit of A FG conversion, consistent with a flattening of the *f_intra,inst_* profile.

### 3.3. Competitive Inter- and Intramolecular Reactions Accounting for Restrictions in Mobility

During polymerization processes, the viscosity of the reaction mixture increases. As a result, the observed reactivity of intermolecular reactions is determined by both the (for simplicity constant) intrinsic rate coefficient and the diffusion rate coefficient, which is depending on the diffusivity of the reactants and their individual FGs. With higher viscosity, the diffusivity at both the molecule and FG level decreases, and the observed (or apparent) reactivity drops. Similarly, intramolecular reactions can be affected as FGs can be too far away from each other due to an increased network rigidity. In other words, apparent kinetics can be active due to diffusional limitations leading to restrictions in mobility, lowering the efficiency of the step-growth polymer network synthesis. In this context, it is worthwhile to theoretically distinguish between the effect of such restrictions on the inter- and intramolecular reaction rates. In what follows this is addressed as effect 1 and effect 2 respectively.

**Figure 10:**
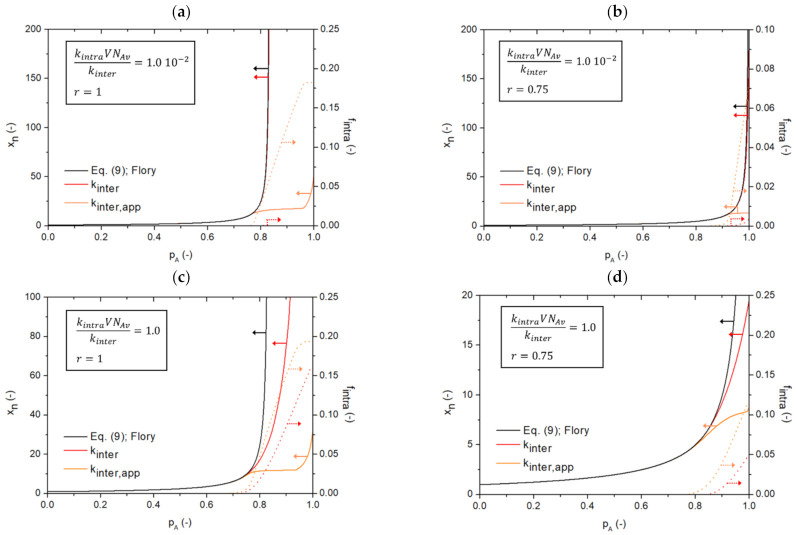
Influence of restricted mobility, and thus, varying (apparent) reactivities for intermolecular reactions (red line vs. orange line) without mobility restrictions for intramolecular reactions for the reaction of an A_3_ monomer and a bifunctional monomer B_2_. Focus is on the effect of the number average chain length *x_n_* as a function of the A FG conversion *p_A_* (left axis; solid lines) for different ratios of $\frac{k_{intra}VN_{Av}}{k_{inter}}$ and different *r* values: (**a**) $\frac{k_{intra}VN_{Av}}{k_{inter}}=1.0\times 10^{-2}$ and *r* = 1, (**b**) $\frac{k_{intra}VN_{Av}}{k_{inter}}=1.0\times 10^{-2}$ and *r* = 0.75, (**c**) $\frac{k_{intra}VN_{Av}}{k_{inter}}=1.0$ and *r* = 1 and (**d**) $\frac{k_{intra}VN_{Av}}{k_{inter}}=1.0$ and *r* = 0.75. Also given are the conventional results according to Equation (9) (Flory equation [22]; black line; no intramolecular reactions). Furthermore, in the same colors, the corresponding variations of the cumulative fraction of intramolecular AB linkages (*f_intra_* variations; right axis; dotted lines); *V*: simulation volume; *N_Av_*: Avogadro number; kinetic Monte Carlo simulations based on Figure 3 (simplification with no mobility restriction for intramolecular reactions; no distance rule).

In Figure 10 it can be seen that diffusional limitations on intermolecular reactions have a huge effect on the molecular structure of the polymer product. For simplicity, it is assumed that intramolecular reactions are still unaffected by diffusional limitations. In the left panel of this figure, emphasis is on *r* = 1 and in the right panel, on *r* = 0.75, in both cases considering the polymer network synthesis between A_3_ and B_2_ monomers (ρ = 1). The top row relates to a low $\frac{k_{intra}VN_{Av}}{k_{inter}}$ value and the bottom row to a large one. The decrease of *k_inter,app_* due to diffusional limitations, compared to the constant/intrinsic *k_inter(,intr)_*, as a function of *p_A_* is given in Figure S8 in the Supplementary Materials. Under stoichiometric conditions (Figure 10a,c): orange solid lines; left axis), it can be seen that at higher A FG conversions, a plateau is established in the *x_n_* profile, meaning that only intramolecular reactions take place as they do not change the number of monomer units per molecule. The number of monomer units per molecule (and thus also *x_n_*) only starts to go up again if no further intramolecular reactions can take place, as *k_inter,app_* still possesses a non-zero value. For the intrinsic case in Figure 10 (red solid lines; left axis) such a plateau is absent, highlighting the relevance of diffusional limitations on intermolecular reactions. For comparison, the Flory [22] equation (Equation (9)) results are also included as full black lines, further demonstrating that they are not representative. Even for a low $\frac{k_{intra}VN_{Av}}{k_{inter}}$ a mismatch results as the plateau is lacking. The existence of a Flory asymptote is thus overruled, which is reflected in the large increase of *f_intra_* (dotted lines; right axis) upon going from the intrinsic to the apparent case (red vs. orange dotted lines; right axis in Figure 10). Similar observations can be made for the non-stoichiometric case (Figure 10b,d): orange solid lines; left axis). The plateau formation is less pronounced and the number of intramolecular linkages less; but despite that, the *x_n_* values are still largely reduced.

**Figure 11:**
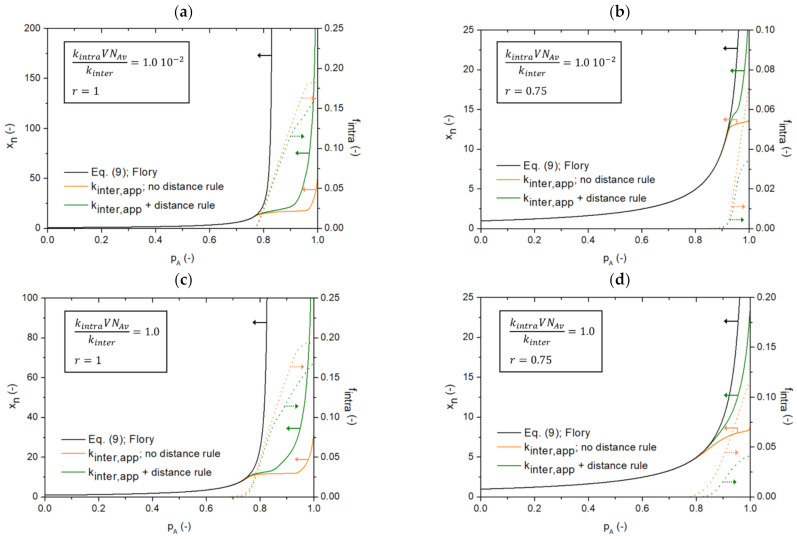
Influence of restricted mobility for intramolecular reactions (orange line vs. green line) for the reaction of an A_3_ monomer and a bifunctional monomer B_2_; same simulation conditions as in Figure 10, thereby also accounting for restricted mobility or varying (apparent) reactivities for intermolecular reactions. Focus is on the effect of the number average chain length *x_n_* as a function of the A FG conversion *p_A_* (left axis; solid lines) for different ratios of $\frac{k_{intra}VN_{Av}}{k_{inter}}$ and different *r* values: (**a**) $\frac{k_{intra}VN_{Av}}{k_{inter}}=1.0\times 10^{-2}$ and *r* = 1, (**b**) $\frac{k_{intra}VN_{Av}}{k_{inter}}=1.0\times 10^{-2}$ and *r* = 0.75, (**c**) $\frac{k_{intra}VN_{Av}}{k_{inter}}=1.0$ and *r* = 1 and (**d**) $\frac{k_{intra}VN_{Av}}{k_{inter}}=1.0$ and *r* = 0.75. Also given are the conventional results according to Equation (9) (Flory equation [22]; black line; no intramolecular reactions). Furthermore, in the same colors, the corresponding variations of the cumulative fraction of intramolecular AB linkages (*f_intra_* variations; right axis; dotted lines); *V*: simulation volume; *N_Av_*: Avogadro number; kinetic Monte Carlo simulations based on Figure 3.

A second effect that needs to be evaluated is that, for a sampled intramolecular reaction to actually happen, the selected FGs need to be sufficiently close to each other. For this, a so-called distance rule is applied in Figure 3 based on the compactness of the environment of the chosen FGs, and thus the local degree of rigidity. In Figure 11, this effect is added to the *k*MC model, starting from the results in Figure 10 without such restrictions for the intramolecular reactions (green lines from detailed model vs. repeated orange lines from Figure 10 without mobility restrictions on intramolecular reactions). It can be seen that this extra restriction in mobility further influences the *x_n_* evolution (left axis) and counteracts the first effect. If intramolecular reactions are affected by the distance rule, intermolecular reactions gain in relative importance, therefore increasing *x_n_* specifically at higher *p_A_*. This is consistent with a decrease for *f_intra_* in Figure 11 (right axis). This decrease is relatively more pronounced under non-stoichiometric conditions (Figure 11b,d), as can also be derived from Figure S9 in the Supplementary Materials, showing the ratio of the cumulative number of failures upon applying the distance rule for reselecting a new molecule to the cumulative number of intramolecular reactions sampled for $\frac{k_{intra}VN_{Av}}{k_{inter}}=1.0$. As long as not enough crosslinking points are formed in the molecules, no intramolecular reactions occur, explaining the zero values at the lower *p_A_* values in Figure S9 in the Supplementary Materials. Under stoichiometric conditions, larger molecules are more easily formed, for which the probability of finding possible intramolecular reacting partners is higher, explaining the trends in Figure S9. In any case, *f_intra_* remains a disturbing factor and thus, to study step-growth kinetics, the competition between inter- and intramolecular reactions needs to be considered.

**Figure 12:**
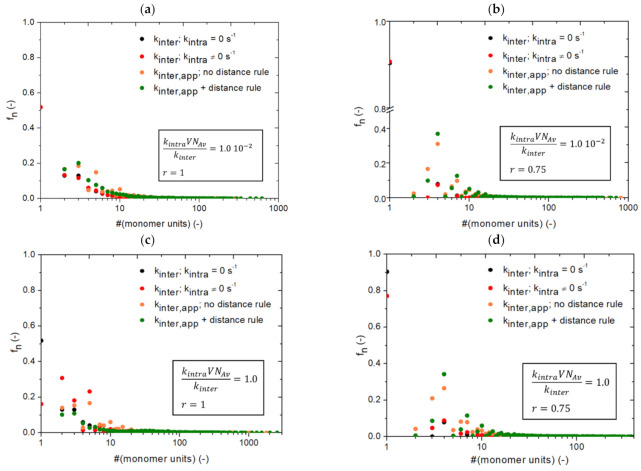
Corresponding number chain length distributions for Figure 10 and Figure 11 at a final *p_A_*: (**a**) $\frac{k_{intra}VN_{Av}}{k_{inter}}=1.0\times 10^{-2}$ and *r* = 1, (**b**) $\frac{k_{intra}VN_{Av}}{k_{inter}}=1.0\times \;10^{-2}$ and *r* = 0.75, (**c**) $\frac{k_{intra}VN_{Av}}{k_{inter}}=1.0$ and *r* = 1 and (**d**) $\frac{k_{intra}VN_{Av}}{k_{inter}}=1.0$ and *r* = 0.75.

It should be realized that the green solid lines, obtained from simulation in Figure 11 are thus closest to an actual synthesis situation, as both restricted mobility for inter- and intramolecular reactions are expected. Again the Flory [22] equation (Equation (9)) is included (black solid lines), and it is further confirmed that its inherent assumptions limit its applicability to the very low *p_A_* region only. In Figure 12, the corresponding chain length distributions (CLDs) for Figure 10 and Figure 11 at a final *p_A_* are shown, differentiation between the ideal Flory case (no intramolecular reactions), the *k*MC case with intramolecular reactions but no mobility restrictions and the two extended cases with diffusional limitations, first for intermolecular reactions only and then additionally for intramolecular reactions. It follows that the starting Flory distributions (black symbols) are perturbed because of intramolecular reactions (black to red symbols), with a shift to the left, but also due to diffusional limitations on intermolecular reactions (red to orange symbols) with again a shift to the left, and ultimately due to diffusional limitations on intramolecular reactions (orange to green symbols), with oppositely a shift to the right. A complex interplay of chemical and diffusion phenomena is therefore observed, leading to a complex shape for the CLD in a real case. To further illustrate the deviations from the traditional equations in Figure S10 in the Supplementary Materials, emphasis is also on a step-growth network synthesis between *A*_3_ and B_3_, considering again *r* = 1 and *r* = 0.75. It can be seen that, again, diffusional limitations have a huge impact on molecular growth and product heterogeneity.

In general, it follows from the theoretical results in Figure 10, Figure 11 and Figure 12 and Figure S10 in the Supplementary Materials that the (experimental) variation of *x_n_* as a function of *p_A_* allows the retrieval of information on the relative importance of inter- and intramolecular reactions. A clear shift beyond the Flory asymptote indicates that intramolecular reactions cannot be ignored. A strong reduction in *x_n_* implies a high (intrinsic) intramolecular reactivity. A clear plateau for *x_n_* further indicates that intramolecular reactions are not strongly affected by mobility restrictions. The consideration of various *r* values facilitates the further validation of the hypothesis made regarding the relative inter- and intramolecular (apparent) reactivities.

## 4. Conclusions

The well-established analytical equations that describe step-growth polymerization in the absence of intramolecular reactions and with equal reactivities, as developed by Flory and Stockmayer, are useful to form an idea of the relation between the maximal functional group (FG) conversion and the functionality degree (*f* value) and level of stoichiometry (*r* value). An associate contour plot, for example, predicts that for *f* = 2, no stoichiometric imbalance is allowed to reach a high FG conversion (*r* = 1), while for *f* = 12, initial compositions with *r* = 0.6 are sufficient to obtain a FG conversion equal to unity. However, in the presence of intramolecular reactions, it is demonstrated that these simplified equations involving asymptotes and ignoring such reactions are biased. As demonstrated using matrix-based kinetic Monte Carlo (*k*MC) simulations, a value of $\frac{k_{intra}VN_{Av}}{k_{inter}}$ equal to 5.0 × 10^−3^, for instance, already introduces an error of 5% on the Flory profile, still assuming constant reactivities. Such *k*MC simulations are a strong tool to fully grasp the interaction between inter- and intramolecular reactions, as they enable the tracking of the connectivity of each separate molecule as a function of time according to kinetic rules.

If mobility restrictions and thus diffusional limitations are relevant, the functional group conversion increases and specifically, the *x_n_* profile is affected. The consideration of stoichiometric and non-stoichiometric conditions allows the identification of not only the actual relative importance in inter- and intramolecular reactivity at the lower reaction times but also at the higher times, at which diffusional limitations kick in.

It has been further shown that reverse engineering allows the testing of mechanistic or modeling hypotheses to improve the understanding of step-growth network synthesis. The present work evaluated two such hypotheses. The first one is the consideration of an average molecule at each functional group conversion, as defined by dividing the total number of A/B functional groups by the number of molecules. This hypothesis proved to be unworkable as a strong relationship exists between the number of intramolecular AB combinations per molecule and the size of the molecule or its number of monomer units. The second one relates to the potential of simplified formulas using dimensionless numbers to describe the instantaneous fraction of intramolecular reactions. It follows that basic formulas are unsuited already in the case of constant reactivities, and preference should be given to the processing of model output directly coming from a detailed matrix-based *k*MC model.

## Data Availability

The data presented in this study are available upon request from the corresponding author.

## Supplementary Materials

The following are available online at https://www.mdpi.com/article/10.3390/polym13152410/s1, Section S1: Additional simulation results for network synthesis, with Figures S1 and S2: Check on numerical convergence of the kinetic Monte Carlo (*k*MC) simulations, Figure S3: Sampling tree, sampling matrix and composite topology matrix for the illustrative system consisting of the molecules M_1_, M_2_ and M_3_, Figure S4: Benchmark between equation derived by Stockmayer (Equation (11) in main text) and the kinetic Monte Carlo (*k*MC) model in the present work without intramolecular reactions and diffusional limitations for network synthesis, Figure S5: Going beyond the Flory and Stockmayer equation for step-growth network synthesis with a multifunctional monomer A_3_ and a bifunctional monomer B_2_ by including intramolecular reactions in the kinetic Monte Carlo (*k*MC) simulations, Figure S6: Comparison between simplified analytical description in the presence of intramolecular reactions assuming that unreacted FGs A and B are evenly distributed over the molecules (integration of Equations (16) and (17) in main text with Maple) and the solution obtained from *k*MC simulations for the number of molecules *N* and the number of unreacted FGs A as function of functional group conversion of A *pA*, Figure S7: Approach towards finding a pseudo-analytical solution to go beyond the Flory equation for step-growth network synthesis with a multifunctional monomer A3 and a bifunctional monomer B2 by including intramolecular reactions in the kinetic Monte Carlo (*k*MC) simulations, Figure S8: *kinter,app* (L mol−1 s−1) as function of the functional group conversion of A pA compared to the intrinsic one, Figure S9: Importance of distance rule, Figure S10: Influence of restricted mobility for intramolecular reactions for the reaction of an A_3_ monomer and a B_3_ monomer and Section S2: Construction of dimensionless parameters *π*_1_ and *π*_2_.
