# Supplementary material for: Going Beyond the Carothers, Flory and Stockmayer Equation by Including Cyclization Reactions and Mobility Constraints
Source: Polymers (Basel). 2021 Jul 22;13(15):2410. doi: 10.3390/polym13152410 (PMC8348631; doi:10.3390/polym13152410)
Supplement: Supplementary file 1 [file polymers-13-02410-s001.zip › polymers-1299360-supplementary.pdf]

Going beyond the Carothers, Flory and Stockmayer Equation by Including Cyclization Reactions and Mobility Constraints

Lies De Keer [1], Paul H. M. Van Steenberge [1], Marie-Françoise Reyniers [1] and Dagmar R. D'hooge [1,2,*]

[1] Laboratory for Chemical Technology (LCT), Ghent University, Technologiepark 125, 9052 Ghent, Belgium; lies.dekeer@ugent.be (L.D.K.); paul.vansteenberge@ugent.be (P.H.M.V.S.); MarieFrancoise.Reyniers@UGent.be (M.-F.R.)

[2] Centre for Textiles Science and Engineering (CTSE), Ghent University, Technologiepark 70a, 9052 Ghent, Belgium

* Correspondence: dagmar.dhooge@ugent.be

S1. Additional simulation results for network synthesis

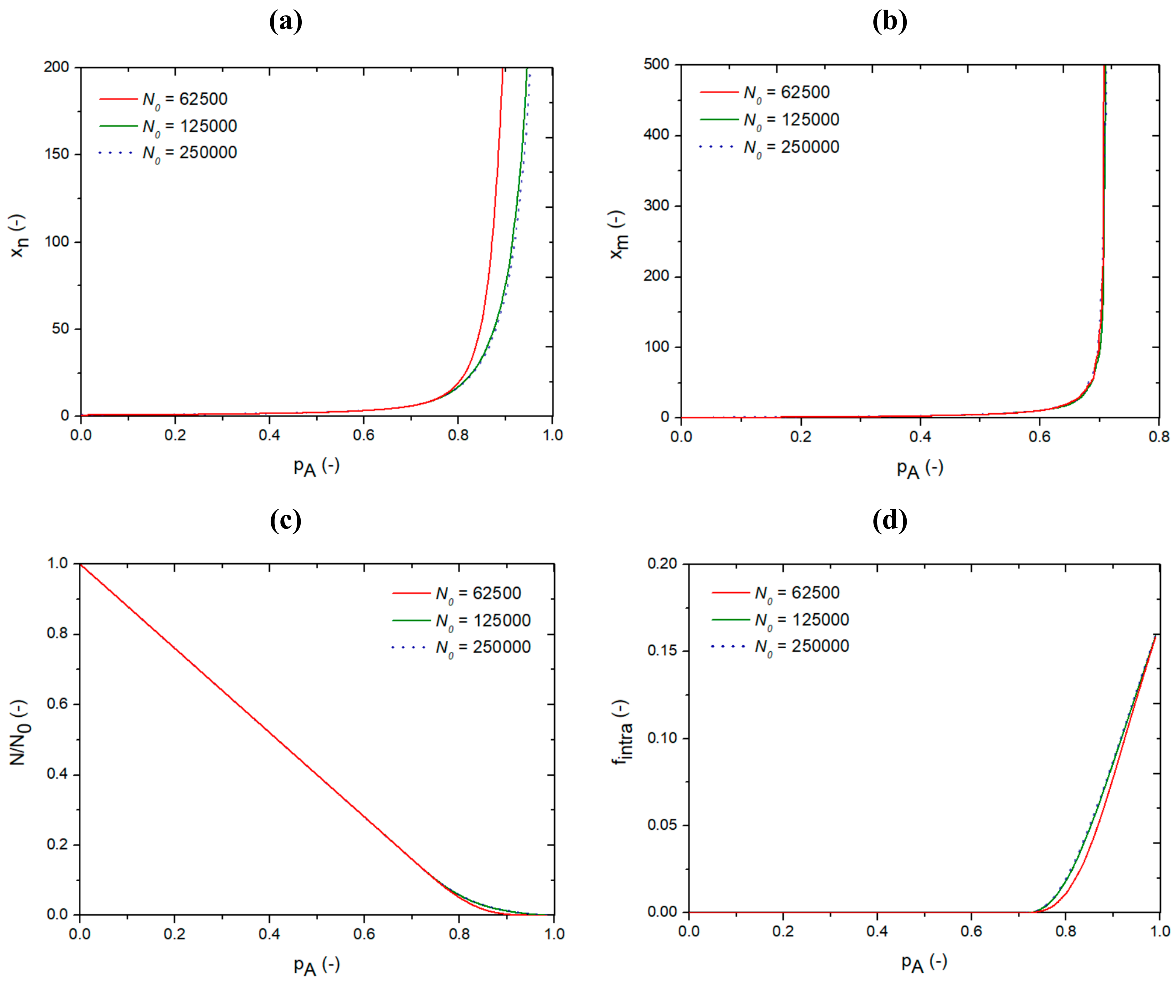

Figure S1. Check on numerical convergence of the kinetic Monte Carlo (*k*MC) simulations. Only if the simulation volume is sufficiently high, representative numerical simulation results are obtained. Examples are given for step-growth network synthesis with a multifunctional monomer A_3 and a

bifunctional monomer B_2 ($\rho = 1$; no mobility restrictions accounted for) for $\frac{k_{intra} V N_{av}}{k_{inter}} = 1.0$: (a) number average chain length x_n as function of the conversion of functional group A p_A, (b) mass average chain length x_m as function of p_A, (c) number fraction of molecules N/N_0 as function of p_A and (d) cumulative fraction of intramolecular AB linkages f_{intra} as function of p_A; simulation results for N_0 = 62500 molecules (V = 5.0 10^{-16} L; red), N_0 = 125000 molecules (V = 1.0 10^{-15} L; green) and N_0 = 250000 molecules (V = 2.0 10^{-15} L; blue).

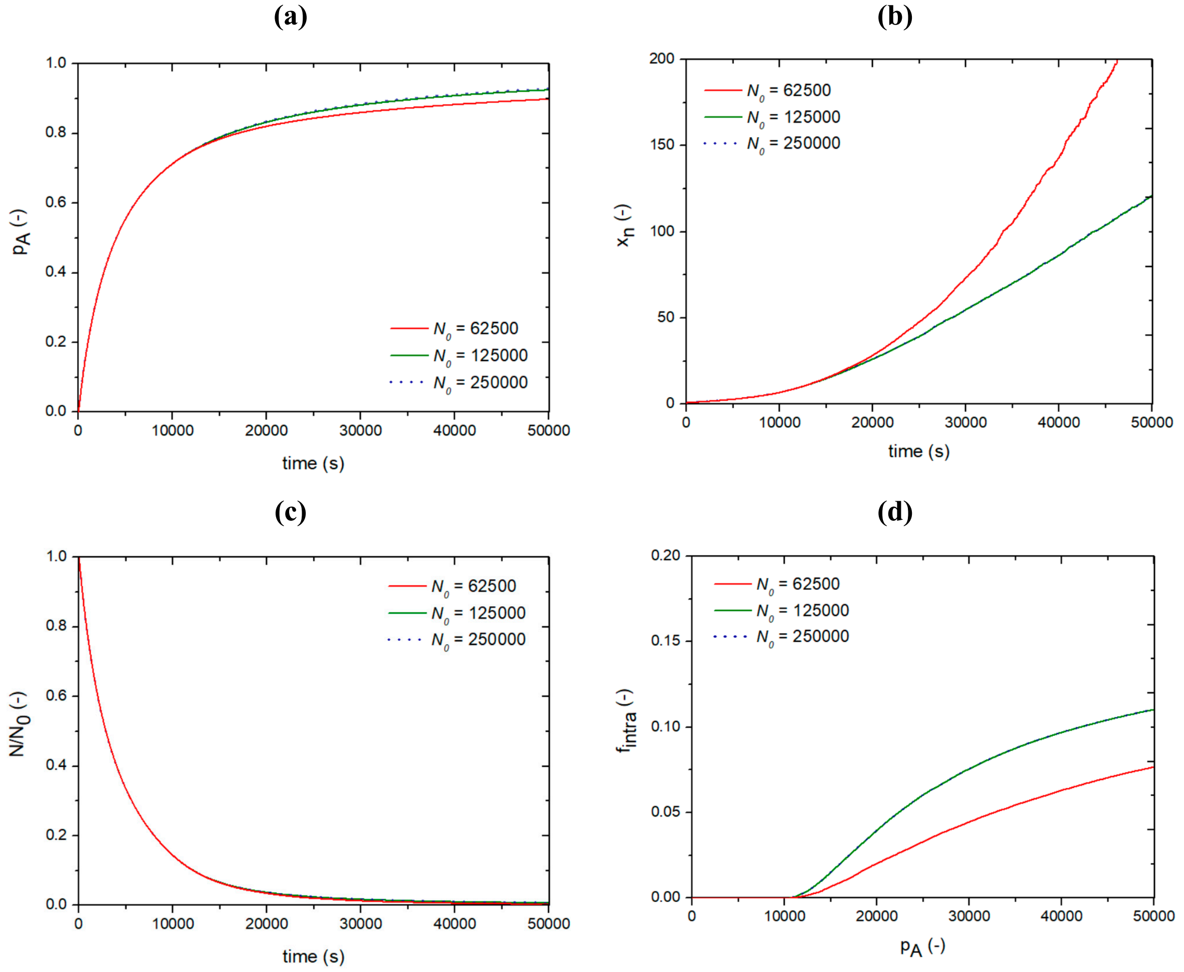

Figure S2. Check on numerical convergence of the kinetic Monte Carlo (*k*MC) simulations. Only if the simulation volume is sufficiently high, representative numerical simulation results are obtained. Examples are given for step-growth network synthesis with a multifunctional monomer A_3 and a bifunctional monomer B_2 ($\rho = 1$; no mobility restrictions accounted for) for $\frac{k_{intra} V N_{av}}{k_{inter}} = 1.0$: (a) conversion of functional group A p_A as function of time (s), (b) number average chain length x_n as function of time (s), (c) number fraction of molecules N/N_0 as function of time (s) and (d) cumulative fraction of intramolecular AB linkages f_{intra} as function of time (s); simulation results for N_0 = 62500 molecules (V = 5.0 10^{-16} L; red), N_0 = 125000 molecules (V = 1.0 10^{-15} L; green) and N_0 = 250000 molecules (V = 2.0 10^{-15} L; blue).

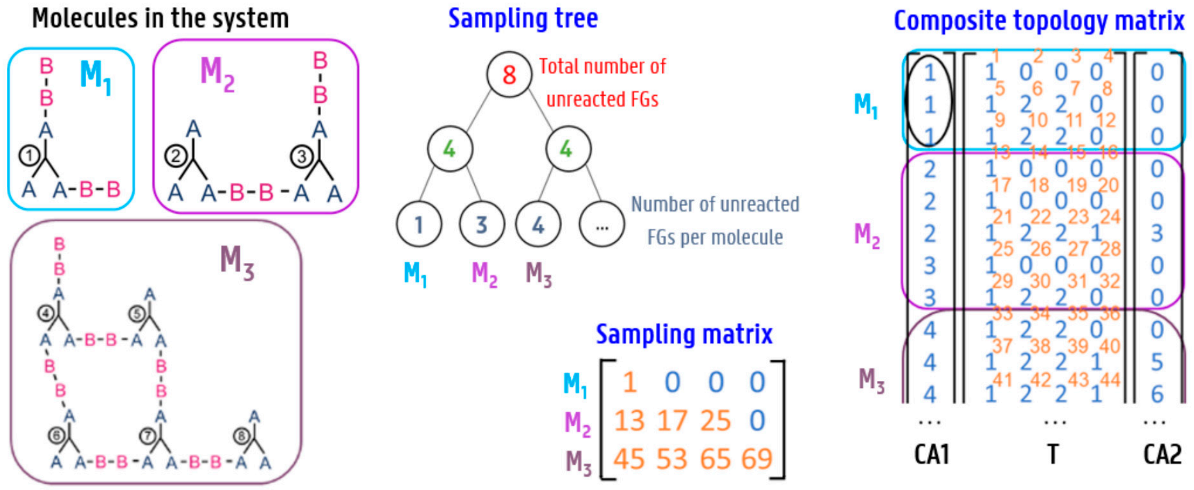

Figure S3. Sampling tree, sampling matrix and composite topology matrix for the illustrative system consisting of the molecules M_1, M_2 and M_3. The composite topology matrix is composed of two connectivity arrays (CA_1 and CA_2) and the topology matrix T (1 and 2 representing functional group A and B respectively. Sampling tree and sampling matrix are shown for functional group A.

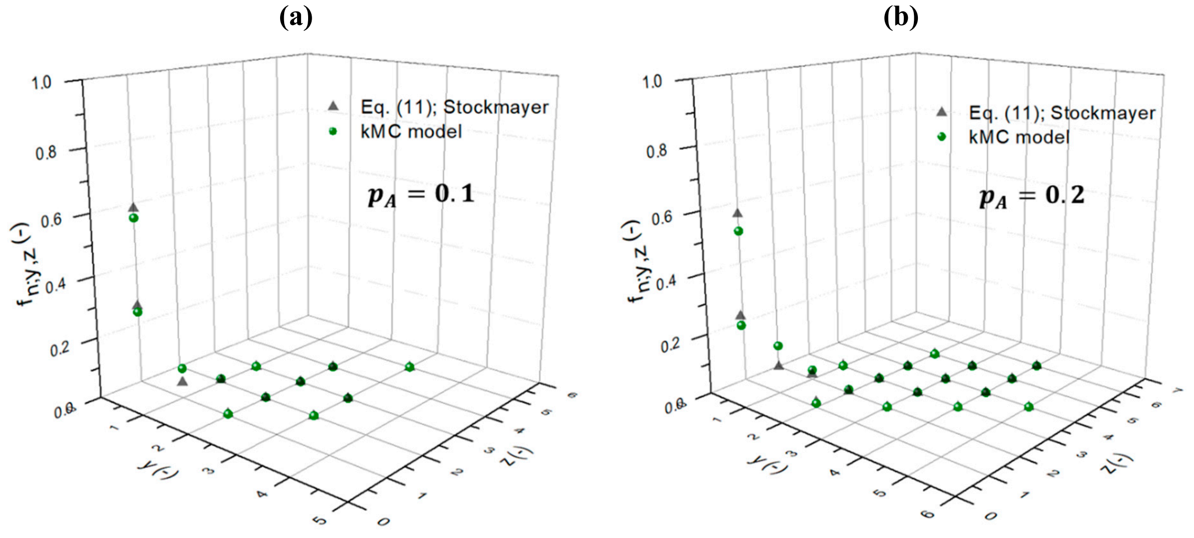

Figure S4. Benchmark between equation derived by Stockmayer [1] (Equation (11) in main text; black) and the kinetic Monte Carlo (*k*MC) model in the present work without intramolecular reactions and diffusional limitations for network synthesis (green), starting from a multifunctional (f = 3; A based) and bifunctional monomer (B_2) and equimolarity of A and B functional groups (r = 1) for $f_{n;y,,z}$ distribution with $f_{n;y,,z}$ representing the number of molecules with y f-functional monomers and z bifunctional monomers incorporated for (a) p_A = 0.1 and (b) p_A = 0.2; complementary to Figure 5 in main text.

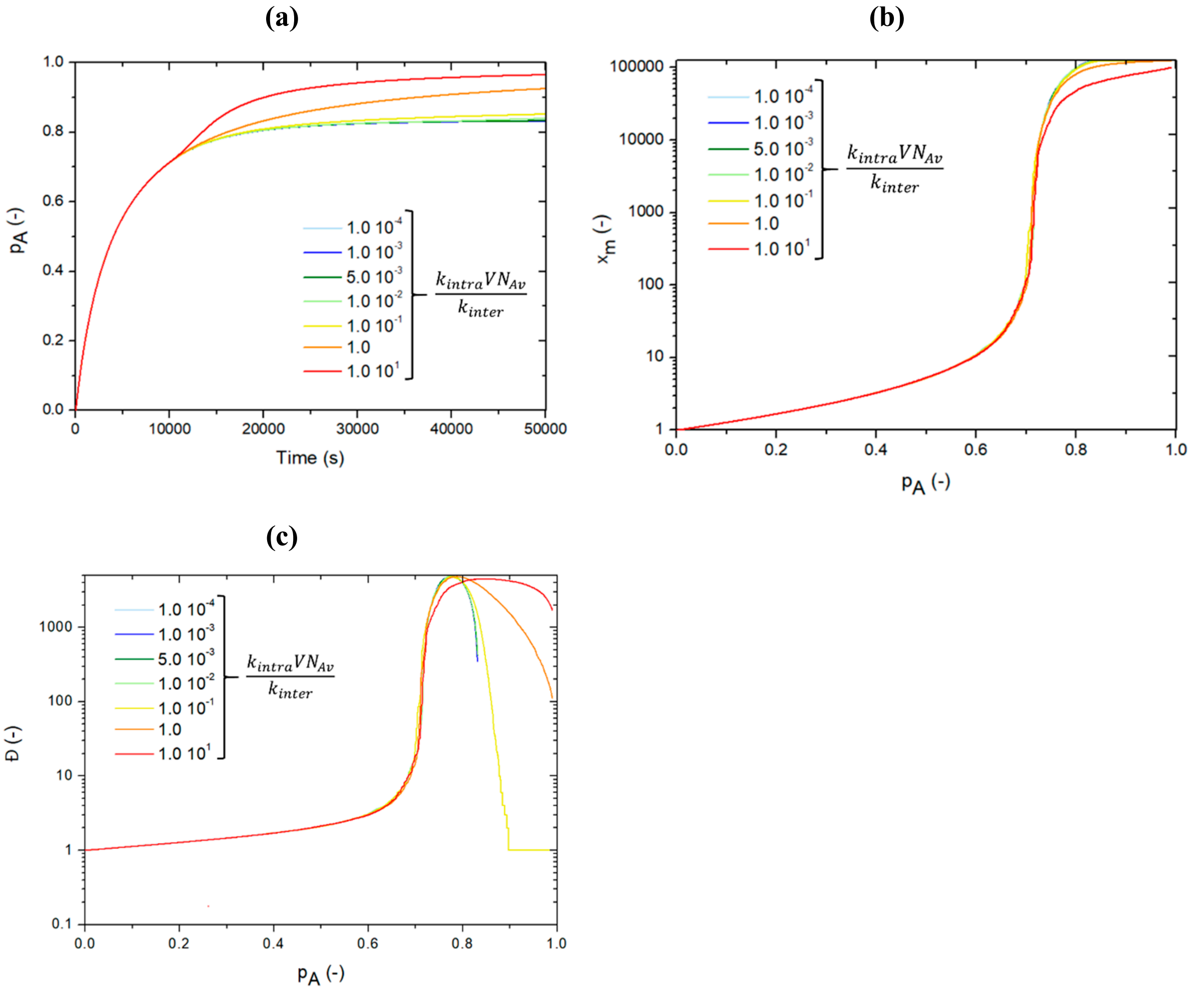

Figure S5. Going beyond the Flory [2] & Stockmayer [3,4] equation for step-growth network synthesis with a multifunctional monomer A_3 and a bifunctional monomer B_2 by including intramolecular reactions in the kinetic Monte Carlo (*k*MC) simulations for the case of equimolarity in A and B functional groups (r = 1) and with still no mobility restrictions or diffusional limitations thus constant reactivities. This done by evaluating the effect of $\frac{k_{intra}VN_{av}}{k_{inter}}$ on (a) the functional group conversion of A (p_A) as function of time (s), (b) the mass average chain length x_m as function of p_A and (c) the dispersity $Đ$ as function of p_A; $k_{\mathrm{intra/inter}}$: intra/intermolecular rate coefficient; V: simulation volume; N_{Av}: Avogadro number; kinetic Monte Carlo simulations based on Figure 3 in main text; complementary to Figure 6 in main text. Note that for one molecule one has dispersity 1.

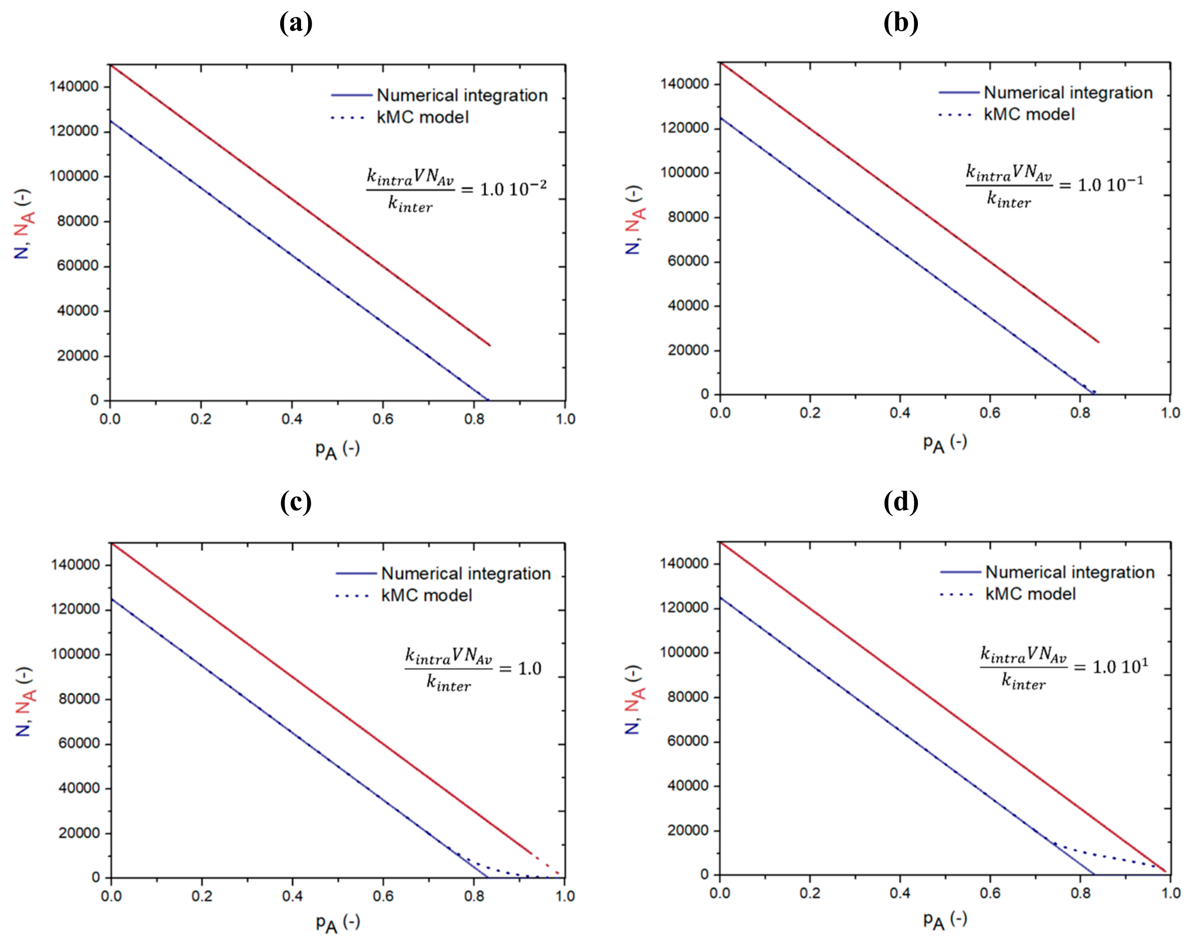

Figure S6. Comparison between simplified analytical description in the presence of intramolecular reactions assuming that unreacted FGs A and B are evenly distributed over the molecules (full lines; integration of Equation (16)-(17) in main text with Maple) and the solution obtained from *k*MC simulations (dotted lines) for the number of molecules *N* (blue) and the number of unreacted FGs A (red) as function of functional group conversion of A p_A for different values of $\frac{k_{intra}VN_{Av}}{k_{inter}}$; no mobility restrictions are accounted for; *V*: volume; N_{Av}: Avogadro number; kinetic Monte Carlo simulations based on Figure 3; complementary to Figure 7 in main text.

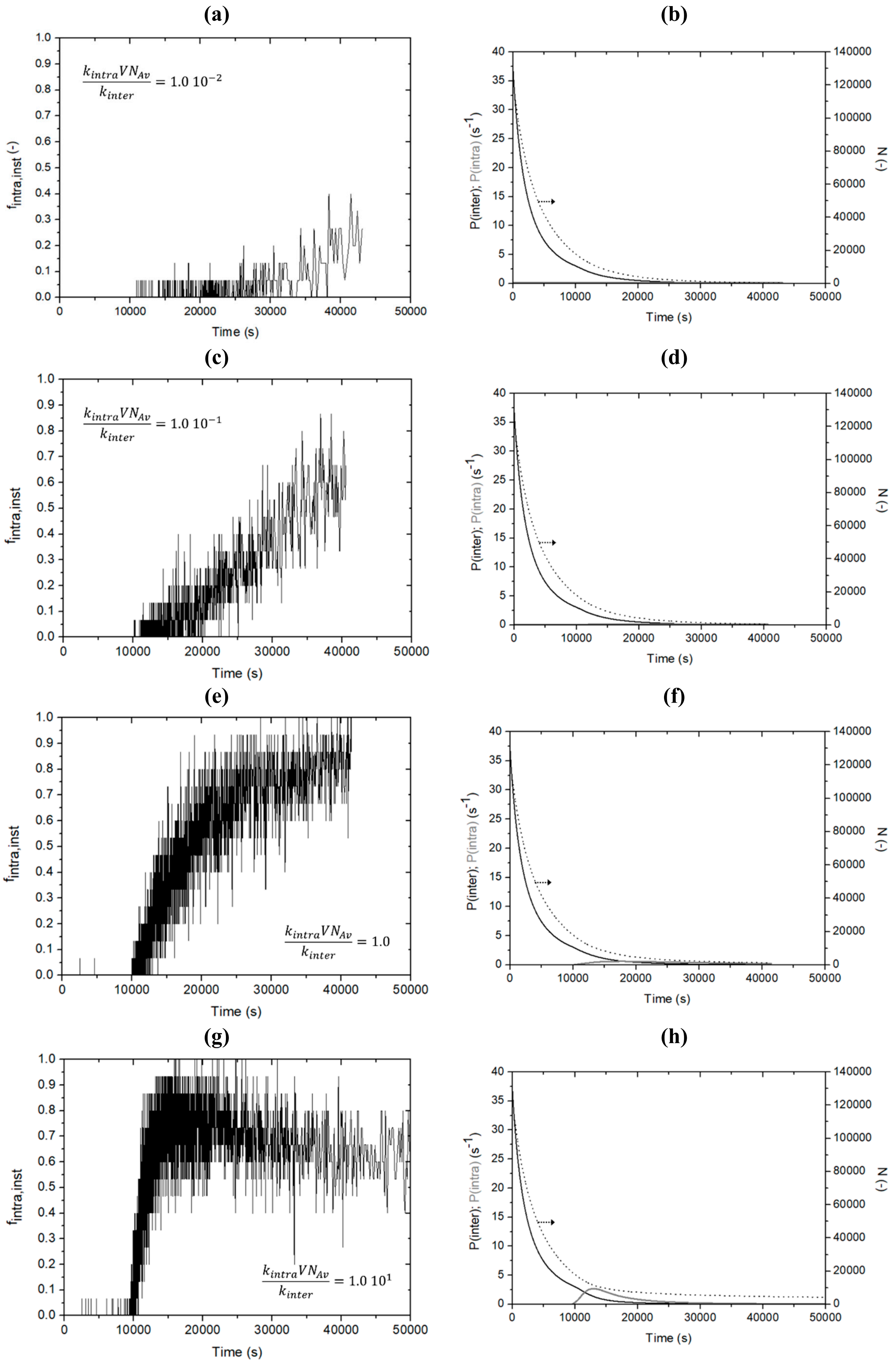
(a)
(b)
(c)
(d)
(e)
(f)
(g)
(h)
$\frac{k_{intra} V N_{Av}}{k_{inter}} = 1.0\ 10^{-2}$
$\frac{k_{intra} V N_{Av}}{k_{inter}} = 1.0\ 10^{-1}$
$\frac{k_{intra} V N_{Av}}{k_{inter}} = 1.0$
$\frac{k_{intra} V N_{Av}}{k_{inter}} = 1.0\ 10^{1}$
$f_{intra,inst}$ (-)
$f_{intra,inst}$
P(inter); P(intra) (s^{-1})
N (-)
Time (s)

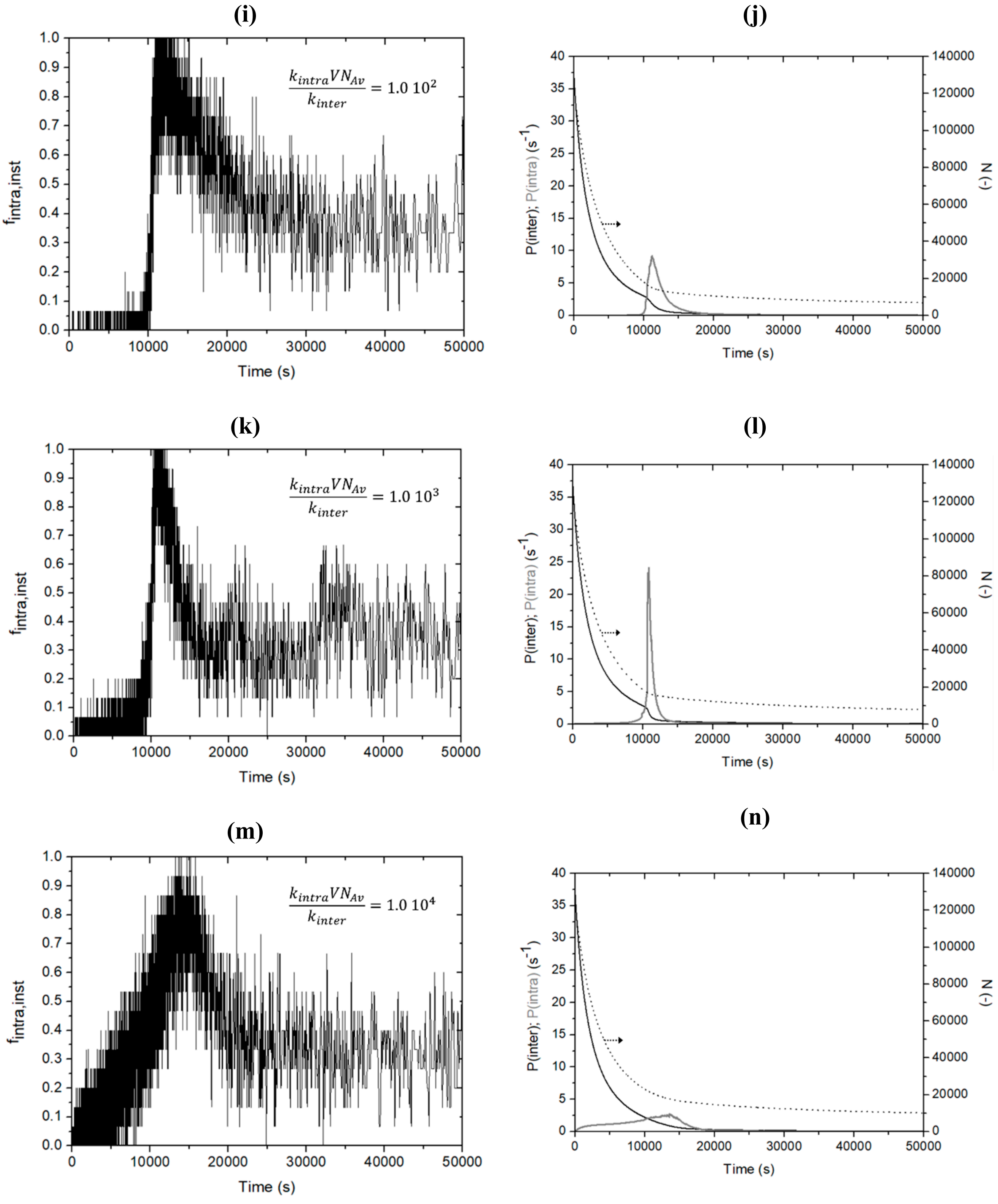

Figure S7. Approach towards finding a pseudo-analytical solution to go beyond the Flory [2] equation for step-growth network synthesis with a multifunctional monomer A_3 and a bifunctional monomer B_2 by including intramolecular reactions in the kinetic Monte Carlo (*k*MC) simulations for the case of equimolarity in A and B functional groups ($r = 1$) and with still no mobility restrictions or diffusional limitations thus constant reactivities: (left) instantaneous fraction of intramolecular reactions $f_{intra,inst}$ as function of time for different values of $\frac{k_{intra}VN_{Av}}{k_{inter}}$ and (right) the corresponding MC rate of intermolecular reaction *P(inter)* (s^{-1}) (left axis; black) and of intramolecular reaction *P(intra)* (s^{-1}) (left axis; grey) as function of time; also given is the number of molecules *N* as function of time (right axis; dotted lines); $k_{intra/inter}$: intra/intermolecular rate coefficient; *V*: simulation volume; N_{Av}: Avogadro number; kinetic Monte Carlo simulations based on Figure 3 in main text; complementary to Figure 9 in main text; note: strictly speaking no complete convergence is obtained for P(inter) and P(intra). Hence,

the predicted $f_{intra,inst}$ is noisy over the complete time range as at low times, not enough FGs exist in the same macromolecules to correctly simulate intramolecular reactivity. Conversely, at higher times, not enough FGs exist in different macromolecules to correctly simulate intermolecular reactivity. Fortunately, this lack of convergence has no kinetic impact.

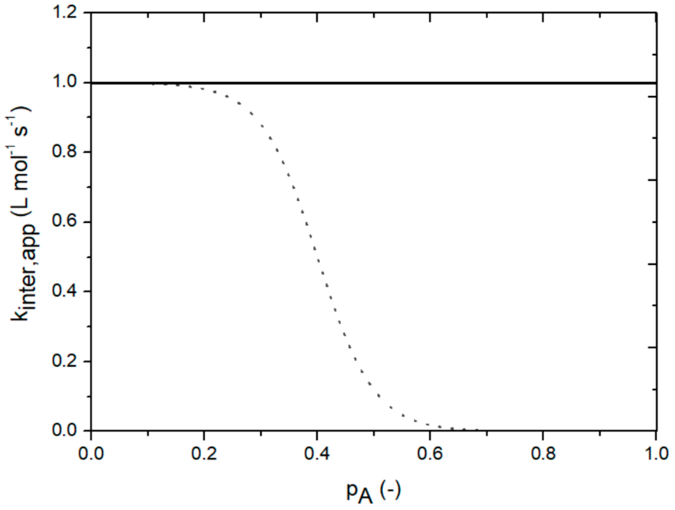

Figure S8. $k_{inter,app}$ (L mol^{-1} s^{-1}) as function of the functional group conversion of A p_A (dotted line) compared to the intrinsic one (full line); complementary to Figure 10-12 in main text.

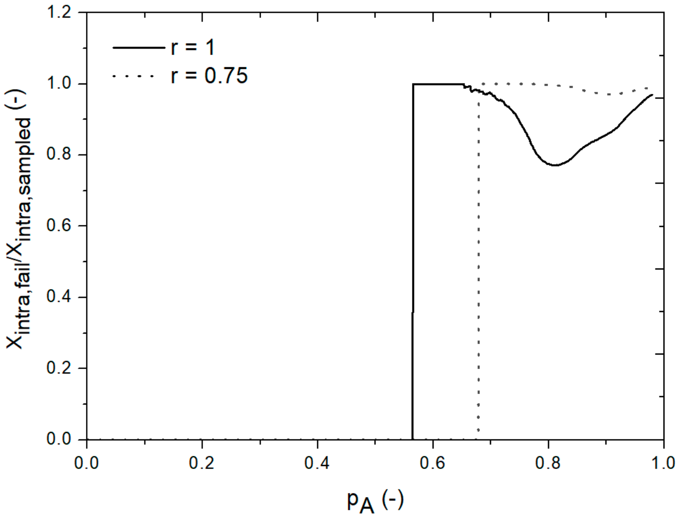

Figure S9. Importance of distance rule: ratio of cumulative number of failed intramolecular reactions $X_{intra,fail}$ due to distance rule to cumulative number of sampled intramolecular reactions $X_{intra,sampled}$ as function of the functional group conversion of A p_A for the reaction of an A_3 monomer and a bifunctional monomer B_2 for $\frac{k_{intra} V N_{av}}{k_{inter}} = 1.0$ and $r = 1$ (full line) compared to $r = 0.75$ (dotted line). Same conditions as in Figure 10 in main text accounting for restricted mobility thus varying (apparent) reactivities for intermolecular reactions; complementary to Figure 10-12 in main text.

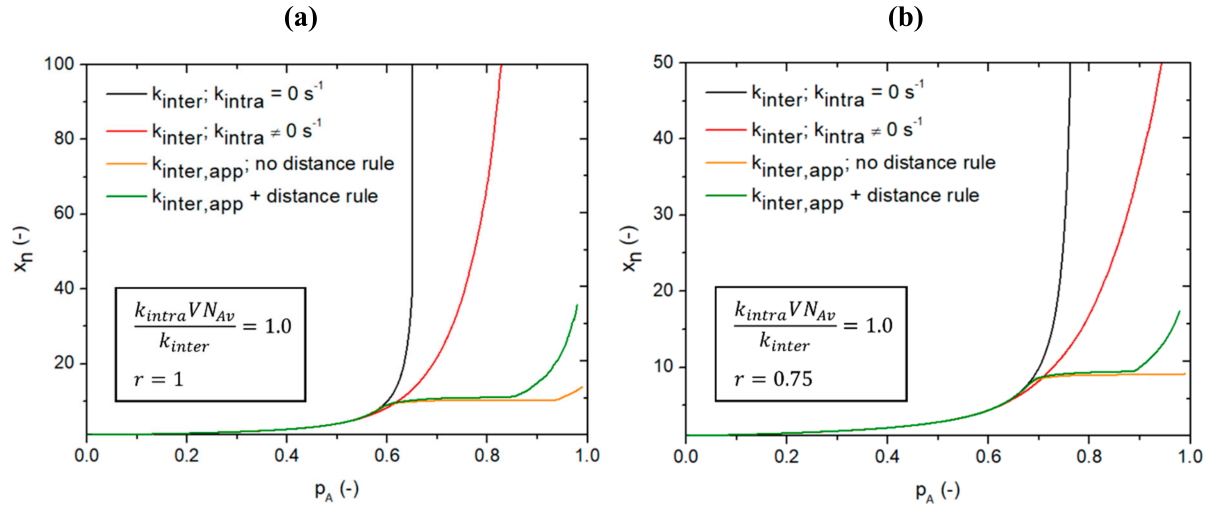

Figure S10. Influence of restricted mobility for intramolecular reactions (dotted orange line vs. full green line) for the reaction of an A_3 monomer and a B_3 monomer for $\frac{k_{intra} V N_{av}}{k_{inter}} = 1.0$: (a) $r = 1$ and (b) $r = 0.75$. Same conditions as in Figure 10 in main text accounting for restricted mobility thus varying (apparent) reactivities for intermolecular reactions; also the associated variations for the cumulative fraction of intramolecular AB linkages (f_{intra} variations) is shown; V: simulation volume; N_{Av}: Avogadro number; kinetic Monte Carlo simulations based on Figure 3; complementary to Figure 11 in main text.

S2. Construction of dimensionless parameters π_1 and π_2

In this section, the functional forms of the dimensionless parameters (π_1 and π_2) as mentioned in the main text are constructed. The proposed starting equations for π_1 and π_2 are given in Equation (S1) and (S2) and are based on the initial reaction rates for intra- and intermolecular reaction respectively, as implemented in the *k*MC model.

$$\pi_1 = k_{intra}[s^{-1}]\left(C_{A,0}C_{B,0}\right)_{same}(VN_{Av})^2 t \tag{S1}$$

$$\pi_2 = k_{inter}[L\ mol^{-1}\ s^{-1}]\left(C_{A,0}C_{B,0}\right)_{diff}(VN_{Av}) t \tag{S2}$$

$C_{A,0}$ and $C_{B,0}$ represent the initial concentrations of FG A and B and can be substituted by the ratio of the number of A and B FGs ($N_{A,0}$ and $N_{B,0}$) and (VN_{Av}) (V: volume (L); N_{Av}: Avogadro's constant (mol^{-1})). For the intramolecular reaction, based on extensive heuristic screening with the *k*MC model, it is found that the initial number of A and B FGs needs to be compensated by subtracting the initial number of molecules N_0, equal to $N_{mon,A,0} + N_{mon,B,0}$.

$$\pi_1 = k_{intra}\left(\frac{N_{A,0} - N_{mon,A,0} - N_{mon,B,0}}{VN_{Av}}\right)\left(\frac{N_{B,0} - N_{mon,A,0} - N_{mon,B,0}}{VN_{Av}}\right)(VN_{Av})^2 t \tag{S3}$$

$$\pi_2 = k_{inter}\left(\frac{N_{A,0}}{VN_{Av}}\right)\left(\frac{N_{B,0}}{VN_{Av}}\right)(VN_{Av}) t \tag{S4}$$

Next, the stoichiometry ratio r (= $N_{A,0}/N_{B,0}$) is introduced to write everything as a function of FG A, as well as the monomer functionalities f_A and f_B using $N_{A,0} = f_A N_{mon,A,0}$. $N_{mon,A,0}$ is written as a function of N_0 using $N_{mon,A,0} = \frac{rf_B}{f_A + rf_B}N_0$.

$$\pi_1 = k_{intra}\left(\frac{rf_Af_B - rf_B - f_A}{f_A + rf_B}N_0\right)\left(\frac{f_Af_B - rf_B - f_A}{f_A + rf_B}N_0\right) t \tag{S5}$$

$$\pi_2 = k_{inter}\left(\frac{1}{VN_{Av}}\right)\left(\frac{rf_Af_B}{f_A + rf_B}N_0\right)\left(\frac{f_Af_B}{f_A + rf_B}N_0\right) t \tag{S6}$$

In the last step, the volume V (L) still present in π_2 and representing the total volume of all FGs, is written as a function of N_0, assuming for simplicity the same molar mass (MM; g mol^{-1}) and density (ρ; g L^{-1}) for FG A and B, thus $MM_{FG,A} = MM_{FG,B} = MM_{FG}$ and $\rho_{FG,A} = \rho_{FG,B} = \rho_{FG}$, as can be seen in Equation (S7).

$$V = V_{FG,A} + V_{FG,B} = \frac{N_{A,0} MM_{FG,A}}{N_{Av}\rho_{FG,A}} + \frac{N_{B,0} MM_{FG,B}}{N_{Av}\rho_{FG,B}} = \left(N_{A,0} + \frac{N_{A,0}}{r}\right)\left(\frac{MM_{FG}}{\rho_{FG}}\right) = \frac{(r+1) f_A f_B}{f_A + r f_B} N_0 \left(\frac{MM_{FG}}{\rho_{FG}}\right) \quad \text{(S7)}$$

Substituting the volume V given by Equation (S7) in Equation (S6) results in the final dimensionless parameters given in Equation (S8) and (S9).

$$\pi_1 = k_{intra} \left(\frac{r f_A f_B - r f_B - f_A}{f_A + r f_B}\right)\left(\frac{f_A f_B - r f_B - f_A}{f_A + r f_B}\right) N_0^2 t \quad \text{(S8)}$$

$$\pi_2 = k_{inter} \left(\frac{MM_{FG}}{\rho_{FG}}\right)^{-1} \left(\frac{r f_A f_B}{(r+1)(f_A + r f_B)}\right) N_0 t \quad \text{(S9)}$$

In Figure S11-S14, simulation results using the *k*MC model, as described in Figure 3 in the main text, are shown, confirming the potential of the functional form of the dimensionless parameters as given in Equation (S8) and (S9). In each figure, a variable in the equations is altered (original simulation: blue line; simulation with altered variable: orange full line) and a dimensionally equivalent simulation result altering another variable is shown (orange dotted line). In each figure except for Figure S11, the rate coefficients are adapted to obtain dimensionally equivalent simulation results as these can be altered independently from each other and have the same proportionality in π_1 and π_2 (k_{intra} directly proportional to π_1 and k_{inter} directly proportional to π_2). The rate coefficients are thus the most straightforward variables to adapt.

For the simulations, a value of 10 mol L^{-1} is used for $\left(\frac{MM_{FG}}{\rho_{FG}}\right)^{-1}$ and $\frac{k_{intra} V N_{Av}}{k_{inter}} = 1$ (other variables are mentioned in the caption of Figure S11-S14). Focus in each of the figures is on the MC rate of the intramolecular reaction *P(intra)* (s^{-1}) (a; left) and the MC rate of the intermolecular reaction *P(inter)* (s^{-1}) (b; right) as a function of time ($f_{intra,inst} = \frac{P(intra)}{P(inter)+P(intra)}$).

Firstly, the rate coefficients k_{intra} and k_{inter} are both multiplied with a factor c (2 in Figure S11).

$$\pi_1 = (k_{intra} * c) \left(\frac{r f_A f_B - r f_B - f_A}{f_A + r f_B}\right)\left(\frac{f_A f_B - r f_B - f_A}{f_A + r f_B}\right) N_0^2 t$$

$$\pi_2 = (k_{inter} * c) \left(\frac{MM_{FG}}{\rho_{FG}}\right)^{-1} \left(\frac{r f_A f_B}{(r+1)(f_A + r f_B)}\right) N_0 t$$

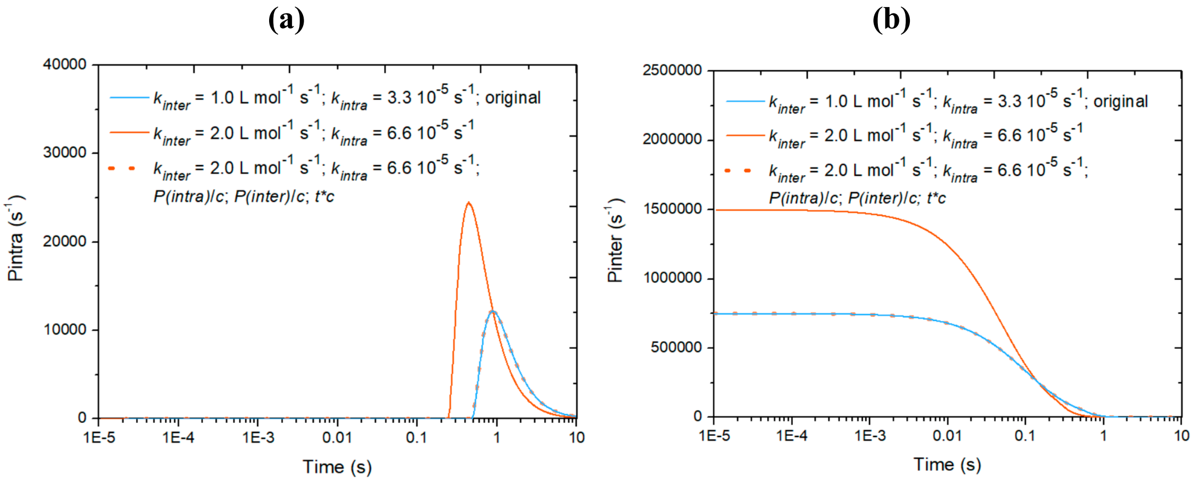

Figure S11. Construction of dimensionless parameters: influence of rate coefficients k_{inter} and k_{intra} (f_A = 3; f_B = 2; r = 1; n_0 = 125000); (a) intramolecular MC rate (s^{-1}) and (b) intermolecular MC rate (s^{-1}) as a function of time; original simulation with k_{inter} = 1.0 L mol^{-1} s^{-1} and k_{intra} = 3.3 10^{-5} s^{-1}: blue line; simulation with altered variables (k_{inter} = 2.0 L mol^{-1} s^{-1} and k_{intra} = 6.6 10^{-5} s^{-1}) resulting in multiplication of π_1 with a factor c = 2 and π_2 with a factor c = 2 and a translation to the left on the time (t) axis with a factor c: orange full line; dimensionally equivalent simulation result altering P(inter), P(intra) and t (P(inter)/c, P(intra)/c and t*c): orange dotted line.

It can be seen in Figure S11 that, as expected, dimensionally equivalent simulation results are obtained by dividing the probabilities with the same factor c. As expected, there is also a translation to the left on the time scale by increasing the rate coefficients, so time needs to be multiplied with c. This result is intuitively clear as any change in rate coefficients will impact the reaction time.

Secondly, the number of molecules N_0 is multiplied by a factor c (0.9 in Figure S12). Figure S12 confirms that dimensionally equivalent simulation results are obtained by dividing k_{intra} by c^2 and k_{inter} by c. This result can intuitively be expected because changing the initial number of molecules changes the number of combinations between FGs in the same or in different macromolecules, which can be counter-acted by changing k_{intra} and k_{inter}.

$$\pi_1 = k_{intra}\left(\frac{rf_Af_B - rf_B - f_A}{f_A + rf_B}\right)\left(\frac{f_Af_B - rf_B - f_A}{f_A + rf_B}\right)(N_0 * c)^2 t$$

$$\pi_2 = k_{inter}\left(\frac{MM_{FG}}{\rho_{FG}}\right)^{-1}\left(\frac{rf_Af_B}{(r+1)(f_A + rf_B)}\right)(N_0 * c)t$$

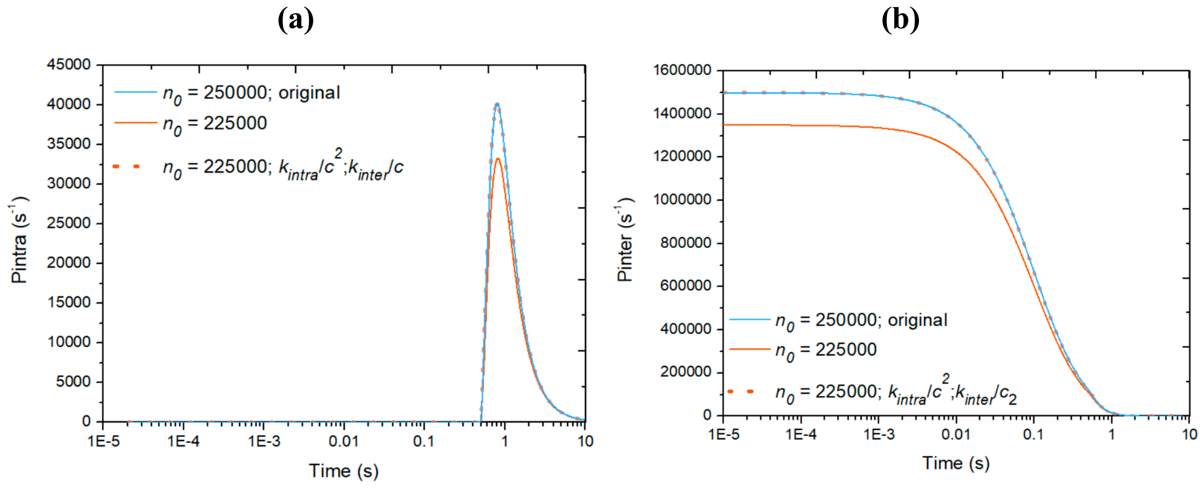

Figure S12. Construction of dimensionless parameters: influence of number of molecules n_0 (f_A = 3; f_B = 2; r = 1); (a) intramolecular MC rate (s^{-1}) and (b) intermolecular MC rate (s^{-1}) as a function of time; original simulation with n_0 = 250000: blue line; simulation with altered variable (n_0 = 225000) resulting in multiplication of π_1 with a factor c^2 = 0.81 and π_2 with a factor c = 0.9: orange full line; dimensionally equivalent simulation result altering k_{intra} and k_{inter} (k_{intra}/c^2 and k_{inter}/c): orange dotted line.

Thirdly, the monomer functionality f_A is altered, leading to a multiplication of dimensionless parameter π_1 with factor c_1^2 (1.2 in Figure S13a) and of dimensionless parameter π_2 with factor c_2 (1.08 in Figure S13b). It can be seen in Figure S13 that dimensionally equivalent simulation results are obtained by dividing k_{intra} and k_{inter} by c_1^2 and c_2 respectively. This result is highly interesting, as it indicates that changes in either k_{intra} or k_{inter} can be counter-acted (to some degree, limited by the discrete nature of the number of FGs in the monomer) by changing the monomer functionality.

$$\pi_1 = k_{intra}\left(\frac{rf_Af_B - rf_B - f_A}{f_A + rf_B} * c_1\right)\left(\frac{f_Af_B - rf_B - f_A}{f_A + rf_B} * c_1\right)N_0^2 t$$

$$\pi_2 = k_{inter}\left(\frac{MM_{FG}}{\rho_{FG}}\right)^{-1}\left(\frac{rf_Af_B}{(r+1)(f_A + rf_B)} * c_2\right)N_0 t$$

(a) (b)

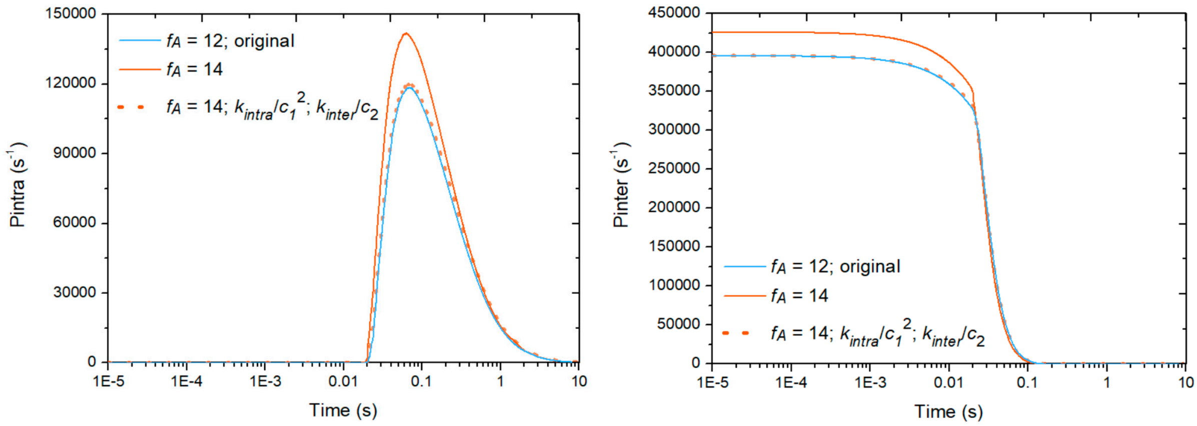

Figure S13. Construction of dimensionless parameters: influence of monomer functionality f_A (f_B = 12; r = 1; n_0 = 13200); (a) intramolecular MC rate (s^{-1}) and (b) intermolecular MC rate (s^{-1}) as a function of time; original simulation with f_A = 12: blue line; simulation with altered variable (f_A = 14) resulting in multiplication of π_1 with a factor $c_1^2 = 1.2$ and π_2 with a factor $c_2 = 1.08$: orange full line; dimensionally equivalent simulation result altering k_{intra} and k_{inter} (k_{intra}/c_1^2 and k_{inter}/c_2): orange dotted line.

Next, the monomer functionality f_B is altered, leading to a multiplication of dimensionless parameter π_1 with factor c_1^2 (1.25 in Figure S14a) and of dimensionless parameter π_2 with factor c_2 (1.1 in Figure S14b). It can be seen in Figure S14 that dimensionally equivalent simulation results are obtained by dividing k_{intra} and k_{inter} by c_1^2 and c_2 respectively. As in the precious case, this result is highly interesting, as it indicates that changes in either k_{intra} or k_{inter} can be counter-acted (to some degree, limited by the discrete nature of the number of FGs in the monomer) by changing the monomer functionality.

$$\pi_1 = k_{intra}\left(\frac{rf_Af_B - rf_B - f_A}{f_A + rf_B} * c_1\right)\left(\frac{f_Af_B - rf_B - f_A}{f_A + rf_B} * c_1\right)N_0^2 t$$

$$\pi_2 = k_{inter}\left(\frac{MM_{FG}}{\rho_{FG}}\right)^{-1}\left(\frac{rf_Af_B}{(r+1)(f_A + rf_B)} * c_2\right)N_0 t$$

(a) **(b)**

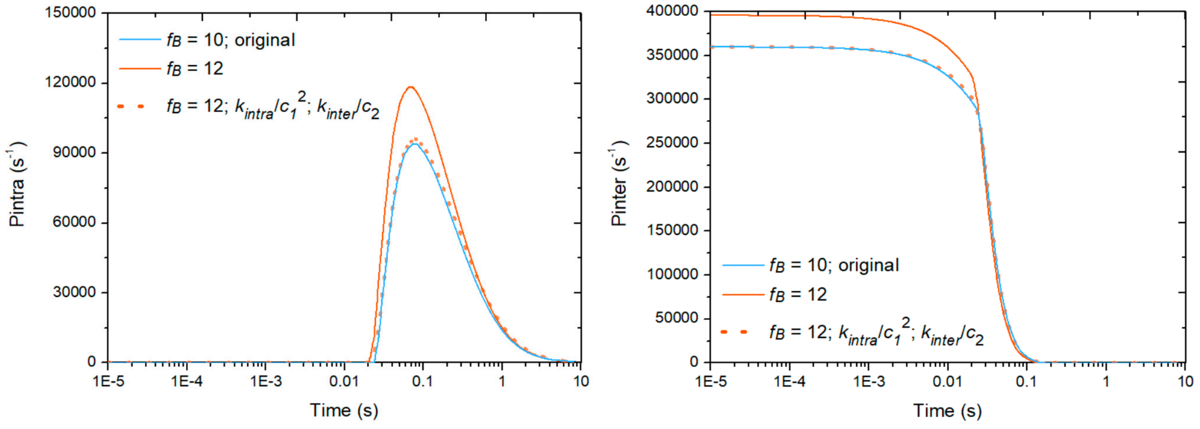

Figure S14. Construction of dimensionless parameters: influence of monomer functionality f_B (f_A = 12; r = 1; n_0 = 13200); (a) intramolecular MC rate (s^{-1}) and (b) intermolecular MC rate (s^{-1}) as a function of time; original simulation with f_B = 10: blue line; simulation with altered variable (f_B = 12) resulting in multiplication of π_1 with a factor $c_1^2 = 1.25$ and π_2 with a factor $c_2 = 1.1$: orange full line; dimensionally equivalent simulation result altering k_{intra} and k_{inter} (k_{intra}/c_1^2 and k_{inter}/c_2): orange dotted line.

Finally, the stoichiometry ratio r is altered, leading to a multiplication of dimensionless parameter π_1 with factor c_1 (0.94 in Figure S15a) and of dimensionless parameter π_2 with factor c_2 (0.97 in Figure S15b). It can be seen in Figure S15 that dimensionally equivalent simulation results are obtained by dividing k_{intra} and k_{inter} by c_1 and c_2 respectively.

$$\pi_1 = k_{intra}\left(\left(\frac{rf_Af_B - rf_B - f_A}{f_A + rf_B}\right)\left(\frac{f_Af_B - rf_B - f_A}{f_A + rf_B}\right) * c_1\right)N_0^2 t$$

$$\pi_2 = k_{inter}\left(\frac{MM_{FG}}{\rho_{FG}}\right)^{-1}\left(\frac{rf_Af_B}{(r+1)(f_A + rf_B)} * c_2\right)N_0 t$$

(a) **(b)**

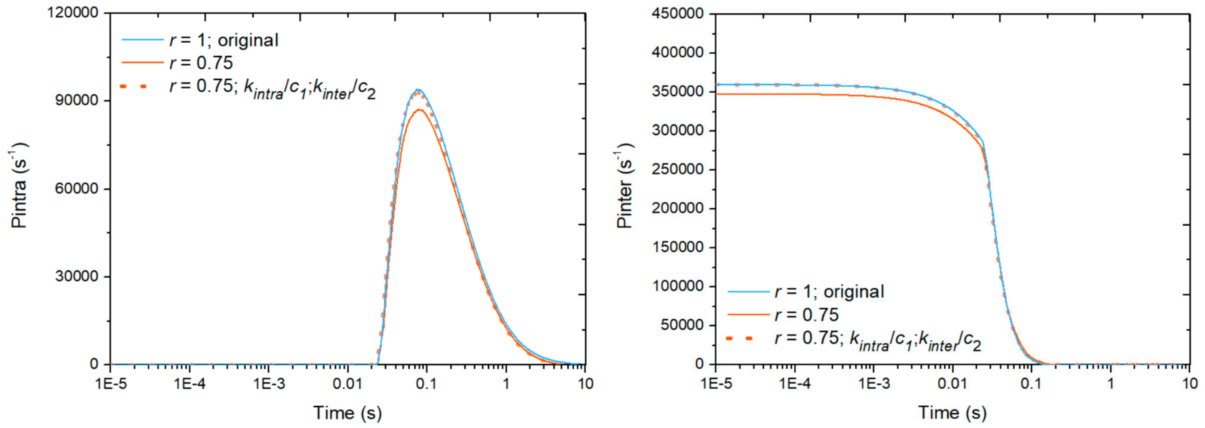

Figure S15. Construction of dimensionless parameters: influence of stoichiometry ratio r (f_A = 12; f_B = 10; n_0 = 13200); (a) intramolecular MC rate (s^{-1}) and (b) intermolecular MC rate (s^{-1}) as a function of

time; original simulation with r = 1: blue line; simulation with altered variable (r = 0.75) resulting in multiplication of π_1 with a factor $c_1 = 0.94$ and π_2 with a factor $c_2 = 0.97$: orange full line; dimensionally equivalent simulation result altering k_{intra} and k_{inter} (k_{intra}/c_1 and k_{inter}/c_2): orange dotted line.

The previous case studies illustrate that certain combinations of synthesis parameters lead to identical MC rates of the inter- and intramolecular reactions. Such combinations can be identified by using the dimensionless parameters π_1 and π_2. This seems to suggest that π_1 and π_2 are two promising dimensionless parameters that determine the competition between inter- and intramolecular reactions, which makes any curve fitting for f_{intra} a much easier task than attempting to correlate it to changes in as much as 6 or 7 synthesis parameters. On the other hand, if diffusional limitations also play a role this correlation is more complex, as also clear from the last part of the main text.

References

1. Stockmayer, W.H. Molecular distribution in condensation polymers. *J. Polym. Sci.* **1952**, *9*, 69-71.
2. Flory, P.J. Molecular Size Distribution in Three Dimensional Polymers. I. Gelation1. *J. Am. Chem. Soc.* **1941**, *63*, 3083-3090.
3. Stockmayer, W.H. Theory of Molecular Size Distribution and Gel Formation in Branched-Chain Polymers. *The Journal of Chemical Physics* **1943**, *11*, 45-55.
4. Stockmayer, W.H. Theory of Molecular Size Distribution and Gel Formation in Branched Polymers II. General Cross Linking. *The Journal of Chemical Physics* **1944**, *12*, 125-131.
